# The costs of scaling up HIV and syphilis testing in low- and middle-income countries: a systematic review

**DOI:** 10.1093/heapol/czab030

**Published:** 2021-03-09

**Authors:** Rabiah al Adawiyah, Olga P M Saweri, David C Boettiger, Tanya L Applegate, Ari Probandari, Rebecca Guy, Lorna Guinness, Virginia Wiseman

**Affiliations:** The Kirby Institute, University New South Wales, High St, Kensington 2052, New South Wales, Australia; The Kirby Institute, University New South Wales, High St, Kensington 2052, New South Wales, Australia; Population Health and Demography, Papua New Guinea Institute of Medical Research, PO Box 60 Homate Street, Goroka, Papua New Guinea; The Kirby Institute, University New South Wales, High St, Kensington 2052, New South Wales, Australia; The Kirby Institute, University New South Wales, High St, Kensington 2052, New South Wales, Australia; Department of Public Health, Faculty of Medicine, Universitas Sebelas Maret, Jl. Ir. Sutami 36A. Surakarta, 57126, Indonesia; The Kirby Institute, University New South Wales, High St, Kensington 2052, New South Wales, Australia; London School of Hygiene and Tropical Medicine, 15-17 Tavistock Place, London WC1H 9SH, UK; Centre for Global DevelopmentEurope, Great Peter House, Great College St, London SW1P 3SE, UK; The Kirby Institute, University New South Wales, High St, Kensington 2052, New South Wales, Australia; London School of Hygiene and Tropical Medicine, 15-17 Tavistock Place, London WC1H 9SH, UK

**Keywords:** Scales, costs, review, healthcare costs

## Abstract

Around two-thirds of all new HIV infections and 90% of syphilis cases occur in low- and middle-income countries (LMICs). Testing is a key strategy for the prevention and treatment of HIV and syphilis. Decision-makers in LMICs face considerable uncertainties about the costs of scaling up HIV and syphilis testing. This paper synthesizes economic evidence on the costs of scaling up HIV and syphilis testing interventions in LMICs and evidence on how costs change with the scale of delivery. We systematically searched multiple databases (Medline, Econlit, Embase, EMCARE, CINAHL, Global Health and the NHS Economic Evaluation Database) for peer-reviewed studies examining the costs of scaling up HIV and syphilis testing in LMICs. Thirty-five eligible studies were identified from 4869 unique citations. Most studies were conducted in Sub-Saharan Africa (*N* = 17) and most explored the costs of rapid HIV in facilities targeted the general population (*N* = 19). Only two studies focused on syphilis testing. Seventeen studies were cost analyses, 17 were cost-effectiveness analyses and 1 was cost–benefit analysis of HIV or syphilis testing. Most studies took a modelling approach (*N* = 25) and assumed costs increased linearly with scale. Ten studies examined cost efficiencies associated with scale, most reporting short-run economies of scale. Important drivers of the costs of scaling up included testing uptake and the price of test kits. The ‘true’ cost of scaling up testing is likely to be masked by the use of short-term decision frameworks, linear unit-cost projections (i.e. multiplying an average cost by a factor reflecting activity at a larger scale) and availability of health system capacity and infrastructure to supervise and support scale up. Cost data need to be routinely collected alongside other monitoring indicators as HIV and syphilis testing continues to be scaled up in LMICs.

**Key Messages**

- Scale is an important driver in determining the costs of HIV and syphilis testing programmes in resource-constrained health systems
- Common methodological assumptions including short-run framework, linear unit cost projections and the availability of health system capacity and infrastructure to supervise expanded delivery currently mask the ‘true’ costs of scaling up HIV and syphilis testing in low- and middle-income countries.
- Financing and budgeting for the scale up of HIV and syphilis testing would benefit from the monitoring of costs across delivery sites over multiple timepoints.

## Introduction

HIV and syphilis infections are major public health problems worldwide (Hook, 2017; Joint United Nations Programme on HIV/AIDS, 2018). In 2017, there were 1.8 million new HIV infections well above the targets set by the World Health Organization (WHO) of <500 000 new infections for HIV per year by 2020 (World Health Organization, 2016; Joint United Nations Programme on HIV/AIDS, 2018). In 2016, an estimated six million new cases of syphilis were reported, far above the WHO target of <600 000 new infections for syphilis per year by 2030 (Joint United Nations Programme on HIV/AIDS, 2018; World Health Organization, 2018). About two-third of all new HIV infections and around 90% of syphilis cases globally occur in low- and middle-income countries (LMICs) (Newman *et al.*, 2013; Joint United Nations Programme on HIV/AIDS, 2018; World Bank, 2019). Untreated HIV and syphilis can lead to mother-to-child transmission (MTCT), which in turn leads to adverse maternal and neonatal outcomes (2018). The global health community, led by the WHO, has identified the dual elimination of MTCT of HIV and syphilis as a public health priority (World Health Organization, 2017a).

Epidemiologically, syphilis has been closely associated with HIV. Both infections have a common route of transmission and syphilitic genital ulcers provide a portal for HIV acquisition (Hook, 2017). Research has long shown that early access to treatment can reduce HIV- and syphilis-related deaths and prevent transmission (Sanders *et al.*, 2005; Bert *et al.*, 2018). However, these benefits are only likely to occur if individuals know their HIV/syphilis status. To facilitate early diagnosis, low-cost, easy to use and highly sensitive and specific HIV and syphilis rapid tests are available (Storey *et al.*, 2019). By the end of 2017, only around 75% of people living with HIV knew their status, and of those diagnosed positive, only 79% received treatment globally (Joint United Nations Programme on HIV/AIDS, 2018). The coverage of syphilis testing lags behind HIV testing in many countries, especially among pregnant women and other high-risk groups such as female sex workers (FSWs) and men who have sex with men (MSM) (Kamb *et al.*, 2010; UNICEF East Asia and Pacific Regional Office, 2016). It has been estimated that, in 2017, only 56% of pregnant women were tested for syphilis in Africa and 31% in South-East Asia (World Health Organization, 2018). In 2019, ∼95% and 57% of pregnant women were tested and treated for HIV in Eastern and Southern Africa and East Asia and Pacific, respectively (UNICEF, 2020). Low testing uptake has led to renewed commitments by national governments and international agencies to scale up HIV and syphilis ‘test and treat’ strategies (Kamb *et al.*, 2010; UNICEF East Asia and Pacific Regional Office, 2016).

Scaling up, broadly defined as the ‘deliberate effort to increase the impact of successfully tested health innovations, so as to benefit more people’ (Simmons and Shiffman, 2007), has a direct impact on costs. Scaling up decentralized tests, outside of laboratories, requires multiple systems to be considered other than the test itself, such as training, quality management, stock managing, reporting and these need to be considered when calculating costs (Johns and Torres, 2005; Mabey *et al.*, 2012). Major divergences have been reported between the costs of HIV and syphilis testing observed in pilot or small-scale studies relative to national roll-out (Johns, 2015; Shelley *et al.*, 2015; Bautista-Arredondo *et al.*, 2018a). Empirical studies have found that this has resulted from the exclusion or inaccurate measurement of the unit costs of transport especially in rural and remote areas, procurement and re-supply of test kits, recruiting and training new staff and strengthening health system infrastructure required for quality control and maintenance (Johns and Torres, 2005; Mikkelsen *et al.*, 2017). Economies of scale also lead to changes in the cost structure of programmes. Theories of economies of scale suggest that the cost per unit decreases as more units are produced (Getzen, 2014). This could be the result of fixed costs being spread over more units of output, reducing per unit costs or due to higher volumes that permit greater specialization of staff (e.g. health facilities in less densely areas may not have enough patient volume for a full-time testing counsellor). In contrast, variable inputs such as test kits and supplies vary directly with the level of output; the amount of fixed inputs cannot be changed in what economists refer to as the ‘short run’. In the short run, the limits of these fixed inputs, whether they are buildings, testing equipment or staffs, imply that further scale up is not possible or will create inefficiencies. In the long run, the fixed inputs are flexible and can be changed to adapt to the different levels of activity, resulting in a non-linear relationship between scale and cost and requiring analysis to distinguish between the short and long runs (Vita, 1990). These relationships and the critical inputs to production will also be different when scaling up services at a clinic to reach more individuals and when scaling up from a single clinic to a national programme (Gomez *et al.*, 2020).

Interest in measuring economies in HIV and syphilis testing of scale has risen with the development of rapid point of care tests (Mabey *et al.*, 2012; Storey *et al.*, 2019). Unit costs of existing automated laboratory testing at a central laboratory may benefit from economies of scale through the distribution of fixed costs, but these costs do not take into account the need for multiple clinic visits, patient costs of accessing testing services and the failure of results to reach patients due to the slow turnaround associated with these tests. Rapid testing at or near the point of care has the potential for savings to the patient through the quick delivery of results and to the provider through a reduction in the number of visits and loss to follow-up (Mabey *et al.*, 2012; Fleming *et al.*, 2017). Levels of testing may also be lower in less densely populated rural areas while the costs of delivery (e.g. cost of the test or transport) can be higher (Shelley *et al.*, 2015).

Currently, decision-makers in LMICs lack a consolidated evidence base from which to understand the cost implications of scaling up HIV and syphilis testing. Three reviews of economic studies evaluating the costs of scaling up of health interventions in LMICs (Kumaranayake *et al.*, 2001; Johns and Torres, 2005) and HIV interventions specifically (Kumaranayake, 2008) have been published, each over a decade old. Each review found evidence of economies of scale, recommending that future work take scale and other cost drivers into account when estimating the costs of scaling up (Kumaranayake *et al.*, 2001; Johns and Torres, 2005; Kumaranayake, 2008). These reviews also identified best practice recommendations for measuring economies of scale and the costs of scaling up public health interventions in LMICs including: ensuring sufficient and representative sample sizes to capture differences in cost characteristics across sites; distinguishing between and measuring both fixed and variable costs; and using appropriate analytical methods, e.g. econometric estimation. None of the reviews capture the recent proliferation of studies assessing the impact of rapid tests for HIV and syphilis. In this paper, we systematically review the current evidence on the costs of scaling up HIV and syphilis testing in LMICs, with a focus on key findings, quality and methodological issues.

## Methods

### Search strategy

The review team defined the search terms according to four domains based on the aims of this review. The four domains included: infectious disease; screening and testing; economics; and LMICs. Despite the frequent use of the term ‘scaling up’ in international health, in practice it has been interpreted in many different ways (Mangham and Hanson, 2010). Search terms relating to ‘scaling up’ were not included in the initial screening, rather an extensive manual screening was conducted to filter articles according to the eligibility criteria listed below. The search terms were applied in six databases: Medline via Ovid; Econlit via Proquest; Embase via Ovid; EMCARE via EBSCO; CINAHL via EBSCO; and Global Health via Ovid. The search strategy used medical subject headings for Medline and comparable terms for the other databases (complete Medline search terms can be found in Supplementary Appendix S1). The NHS Economic Evaluation Database, a bibliographic record of published health technology assessments, was also searched using the same search strategy. A librarian from the authors institute was consulted on the search strategy including the selection of domains and search terms. The systematic review followed the Preferred Reporting Items for Systematic Reviews and Meta-Analyses (PRISMA) guidelines (Moher *et al.*, 2009) and is registered on the International Prospective Register of Systematic Review (PROSPERO), identification number CRD42018103890.

### Eligibility criteria

Studies were eligible for this review if they: (i) measured the costs of scaling up HIV and/or syphilis testing at scale using empirical data, modelling or a hybrid of these approaches; (ii) focused on HIV and/or syphilis testing to identify new infections; (iii) were conducted in an LMICs, defined according to the World Bank Country and Lending Groups classification in 2019 (World Bank, 2019); and (iv) were full research papers (reviews, editorials, letters and conference papers were excluded). No date or language restrictions were applied.

### Study selection

The search results were imported into an Endnote library and were independently screened by two reviewers based on their titles and abstracts. Both reviewers screened the first 10% of articles (*N* = 487) against the eligibility criteria to determine the inter-rater reliability of the reviews. Agreement was assessed using a simple kappa analysis (McHugh, 2012) and near perfect agreement (kappa score of 0.96) was achieved and subsequently the screening continued with a single reviewer (RA) (McHugh, 2012; Wiseman *et al.*, 2016). Two reviewers read the full text of the selected studies and any disagreements regarding eligibility were resolved by consulting a third reviewer. Reasons for exclusion were recorded. Scopus-Elsevier was used to track the reference lists in the final papers to identify any additional relevant studies.

### Data extraction and appraisal

The variables used to describe the different studies in this review are shown in Table 1. All data were extracted by two reviewers and all differences were again resolved by a third reviewer. A narrative synthesis describing the included studies and their conclusions was considered to be the most appropriate approach to synthesizing the findings of the studies (Ryan and Cochrane Consumer and Communication Review Group, 2013). The synthesis from data extraction is presented according to the characteristic of studies included, type of cost studies undertaken, methods to assess cost and impact of scale on costs.

**Table 1:** List of data extraction variables

Study characteristics	

Intervention(s)	Type of HIV and/or syphilis testing activity or programme
Country	Location of study
Study population	Group targeted by intervention(s)
Setting	Service through which the intervention is delivered (e.g. health centre, hospital) and sector (e.g. public/private)
Time horizon	The duration over which costs and/or consequences are calculated
Study design	Randomized controlled trial, cross-sectional, cohort, case–control, modelling
Type of economic analysis (and ratio if applicable)	Cost analysis, cost‐effectiveness, cost‐utility or cost–benefit analysis. Includes ratio used (e.g. cost per DALY averted)
Data source(s)	Primary data collection, expert/stakeholder opinion, published data or literature or combination of those
Analytical approach to measure costs at scale	Econometric, empirical, modelling or a hybrid of these approaches (Kumaranayake, 2008)

Costs of scaling up	

Definition of scaling up	As described by authors
Year (costs)	Year of currency values presented (e.g. 2018 dollars)
Unit(s) of output	Choice of output measure (e.g. number of clients tested, number of facilities with testing available)
Sample size	Total number of, e.g facilities, individuals, tests
Timeframe for decision	Short run (fixed inputs cannot be changed) and long run (all inputs can be changed) (Kumaranayake, 2008)
Cost categories	Categorization of costs as defined by author(s)
Economies/diseconomies of scale	How costs changed with scale of output and by how much.
Empirical results	Specific findings related to the costs of scaling up (e.g. coefficients of scale)
Key drivers of costs identified	Key drivers of the costs of scaling up (e.g. geography, population sub-group, type of providers)

The reporting and methodological quality of studies estimating cost at scale were assessed using a checklist designed for this review (Table 2). The checklist was developed in consultation with global experts in the development of existing checklists for costing exercises and include a checklist for good practice in the economic evaluation of health interventions (Husereau *et al.*, 2013); checklists for appraising priority setting studies in the health sector (Peacock *et al.*, 2007; Wiseman *et al.*, 2016); Global Health Cost Consortium guiding principles (Vassall *et al.*, 2017); and best practice guidelines for calculating the costs of scaling up health interventions (Johns and Baltussen, 2004; Johns and Torres, 2005; Kumaranayake, 2008). Three reviewers independently appraised the studies against this checklist, and any disagreements were resolved by consulting two additional reviewers.

**Table 2:** List of appraisal checklist questions

Standard of reporting costs		

#1	Was the research question(s) well defined?	(Yes/No/Partially addressed)
#2	Was the perspective of the cost estimation clearly stated?	(Yes/No/Partially addressed)
#3	Was the time horizon of sufficient length to capture the costs of the intervention at scale?	(Yes/No/Partially addressed)
#4	Did the study include relevant inputs in the cost estimation (i.e. consumables, human resources, equipment and infrastructure, and managerial practice) (Johns and Torres, 2005)?	(Yes/No/Partially addressed)
#5	Were the methods for estimating the quantities of inputs clearly described?	(Yes/No/Partially addressed)
#6	Did the study clearly report the selection of data source(s) for the ‘units’ estimated in the cost per unit?	(Yes/No/Partially addressed)
#7	Was the sample size determined by the precision required for costing? If not, was the sample designed to be an accurate representation of the study population?	(Yes/No/Partially addressed)
#8	Did the study use relevant and appropriate discount, inflation, and currency conversion rates to enable cost adjustment over setting and time?	(Yes/No/Partially addressed)
#9	Did the study perform sensitivity analyses to characterize uncertainty associated with cost estimates?	(Yes/No/Partially addressed)
#10	Were cost estimates reported and communicated in a clear and transparent way?	(Yes/No/Partially addressed)

Methodological quality of estimating costs at scale		

#11	Were average costs estimated for different levels of scale; and if so, did the study account for changes in input costs associated with increasing scale of the intervention?	(Yes/No/Partially addressed)
#12	Did the study quantify the relationship between average cost and scale?	(Yes/No/Partially addressed)
#13	Were fixed and variable costs analysed separately as scale increased?	(Yes/No/Partially addressed)
#14	Were factors other than scale, that could impact on cost, accounted for in the analysis? (e.g. scope, geography, target population, type of provider)	(Yes/No/Partially addressed)

## Results

**Figure 1:**
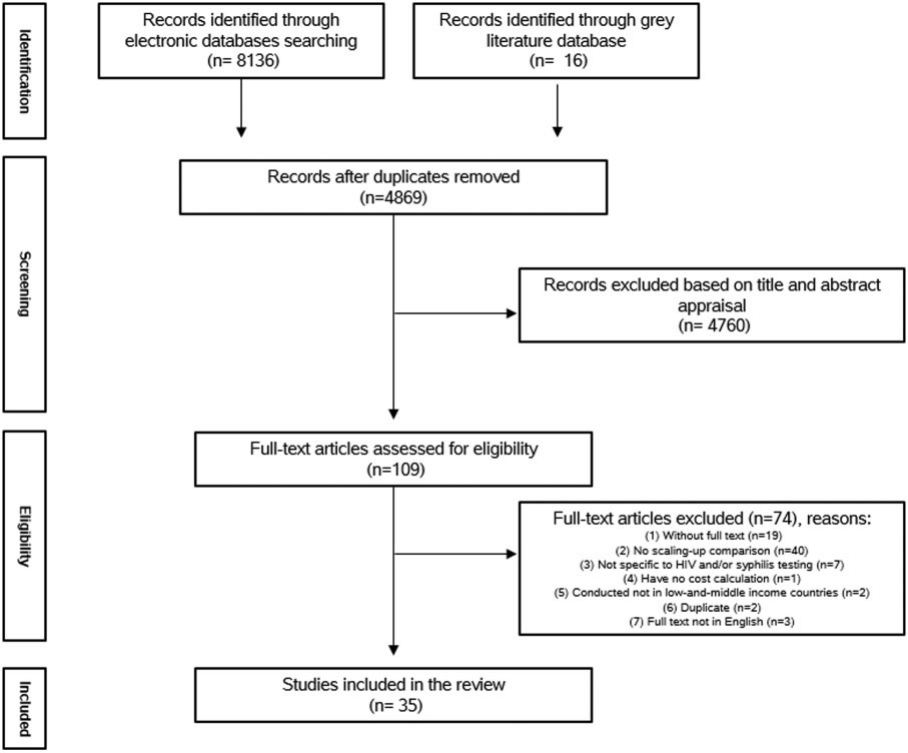
PRISMA flow diagram

We identified 35 eligible studies from 4869 unique citations as shown in the PRISMA flow diagram (see Figure 1).

### Characteristics of the studies included

Table 3 summarizes the characteristics of 35 included studies in this review. The majority of studies were conducted in Sub-Saharan Africa (*N* = 17), published after 2010 (*N* = 24) and focused on HIV testing (*N* = 33). Only two studies examined the costs of scaling up syphilis testing (Schackman *et al.*, 2007; Shelley *et al.*, 2015). Most of the HIV studies involved facility-based testing using rapid tests in the general adult population (*N* = 19) or among pregnant women (*N* = 6), while all syphilis studies were focused on pregnant women (*N* = 2). Only three studies explored the use of HIV self-testing in the community and general population (*N* = 3) (Cambiano *et al.*, 2015; McCreesh *et al.*, 2017; Mangenah *et al.*, 2019). Four studies evaluated community-based testing (Tromp *et al.*, 2013; McCreesh *et al.*, 2017; Cherutich *et al.*, 2018; Mangenah *et al.*, 2019), only one on home-based testing (Sharma *et al.*, 2016), one on mobile testing (Verstraaten *et al.*, 2017) and one in the prison (Nelwan *et al.*, 2016). ‘Scale up’ was most commonly defined in terms of an increase in population coverage (*N* = 32) with the remaining studies defining it as an increase in the number of test kits distributed (*N* = 2) or an expansion of a geographical catchment area (*N* = 1).

**Table 3:** Studies of the cost of scaling up HIV and syphilis testing

Study	Intervention(s)	Country	Study population	Setting	Time horizon	Study design	Type of economic analysis (and ratio if applicable)	Data source (s)	Analytical approach to measure scale
#1 (Shelley *et al.*, 2015)	Syphilis testing/RST/POC	Zambia	Pregnant women (age not specified)	ANC clinic	5 months	Cross-sectional study	Cost analysis	Primary data collection	Empirical
#2 (Schackman *et al.*, 2007)	Syphilis testing, comparing: #1 Syndromic surveillance/not POC #2 RPR for syphilis/not POC #3 RST/POC	Haiti	Pregnant women (age not specified)	ANC clinic	Not specified	Modelling	Cost-effectiveness analysis	Published literature/data	Modelling
#3 (Bautista-Arredondo *et al.*, 2018a,b)	HIV voluntary and provider-initiated counselling and testing/HIV rapid test/not POC	Nigeria	General population (age not specified)	Health facility	6 months	Cross-sectional study	Cost analysis	Primary data collection	Econometric
#4 (Dandona *et al.*, 2008a,b)	HIV voluntary counselling and testing/HIV rapid test/not POC	India	General population (age not specified)	Health facility	1 year	Cross-sectional study	Cost analysis	Primary data collection	Empirical
#5 (Galárraga *et al.*, 2017)	HIV testing and counselling/HIV rapid test/not POC	Kenya	General population (age not specified)	Health facility	22 months	Cross-sectional study	Cost analysis	Primary data collection	Econometric
#6 (Hontelez *et al.*, 2013)	HIV testing/type of test not specified/not POC, comparing: #1 ART at CD4 count ≤350 cells/µl #2 Universal testing and treatment	South Africa	General population (aged 15–65 years)	Not specified	Lifetime	Modelling	Cost-effectiveness analysis	Published literature/data	Modelling
#7 (Ishikawa *et al.*, 2016)	HIV testing/HIV rapid test/not POC, comparing: #1 The current coverage #2 A focused approach #3 A universal approach	Namibia, Kenya, Haiti and Vietnam	Pregnant women (aged 15–49 years old)	ANC clinic	20 years	Modelling	Cost-effectiveness analysis	Published literature/data	Modelling
#8 (McConnel *et al.*, 2005)	HIV rapid voluntary counselling and testing/HIV rapid test/POC	South Africa	General population (age not specified)	VCT clinic	1 year	Cross-sectional study	Cost analysis	Primary data collection	Empirical
#9 (Ahaibwe and Kasirye, 2013)	HIV testing/HIV rapid test/not POC, comparing: #1 The current coverage #2 100% coverage of adult population	Uganda	General population (adult 15–49 years)	Stand alone, integrated w/health facility, non- health facility and mobile VCT services	Lifetime	Modelling	Cost-effectiveness analysis	Published literature/data	Modelling
#10 (Alsallaq *et al.*, 2017)	HIV testing/HIV rapid test/not POC, comparing: #1 HIV strategies focusing on youth (15–24 years old) #2 HIV strategies focusing on adults (15+ years)	Kenya	Youth population (aged 15–24 years)	Health facility	20 years	Modelling	Cost-effectiveness analysis	Published literature/data	Modelling
#11 (Cambiano *et al.*, 2015)	HIV testing/HIV rapid test/not POC, comparing: #1 Provider-delivered HIV testing and counselling #2 HIV self-testing	Zimbabwe	General population (aged 15–65 years old)	Not specified	20 years	Modelling	Cost-effectiveness analysis	Published literature/data	Modelling
#12 (Cherutich *et al.*, 2018)	aPS HIV testing/rapid HIV test/not POC, comparing: #1 Current coverage at 5% #2 Scale up to reach coverage of 50%	Kenya	General population (age not specified)	Health facility and community-based health services	5 years	Modelling	Cost-effectiveness analysis and budget impact analysis	Published literature/data	Modelling
#13 (Mwenge *et al.*, 2017)	HIV voluntary and provider-initiated counselling and testing/rapid HIV test/not POC	Malawi, Zambia and Zimbabwe	General population (aged 15–49 years)	Health facility	1 year	Cross-sectional study	Cost analysis	Primary data collection	Econometric
#14 (Stuart *et al.*, 2018)	HIV testing/type of test not specified/not POC	South Africa	General population (age not specified)	Not specified	30 years	Modelling	Cost analysis	Published literature/data	Modelling
#15 (Tromp *et al.*, 2013)	HIV voluntary counselling and testing/HIV rapid test/not POC, comparing: #1 Current practice #2 Scaling up to reach coverage 80%	Indonesia	Key populations (FSWs, IDUs, higher-risk MSM, transgender, prisoner, clients of FSWs and partner IDUs) (age not specified)	Community-based VCT clinic	20 years	Modelling	Cost-effectiveness analysis	Published literature/data	Modelling
#16 (Zhuang *et al.*, 2018)	HIV testing and treatment/type of test not specified/not POC, comparing: #1 Current strategy #2 Reached 90–90–90 target by 2020 # Reached 90–90–90 target by 2025	China	MSM (age not specified)	Health facility	20 years	Modelling	Cost-effectiveness analysis	Published literature/data	Modelling
#17 (Dandona *et al.*, 2008a,b)	HIV counselling and testing/HIV rapid test/not POC	India	Pregnant women (age not specified)	Hospital and community health centre	1 year	Cross-sectional study	Cost analysis	Primary data collection	Econometric
#18 (Dandona *et al.*, 2005)	HIV counselling and testing/HIV rapid test/not POC	India	General population (age not specified)	VCT clinics	1 year	Cross-sectional study	Cost analysis	Primary data collection	Econometric
#19 (Forsythe, 2002)	HIV voluntary counselling and testing/serial of HIV rapid testing/POC	Kenya	General population (age not specified)	Health facility	A year	Cross-sectional study	Cost analysis	Primary data collection	Empirical
#20 (Granich *et al.*, 2009)	HIV testing/type of test not specified/not POC, comparing: #1 Reference scenario #2 Universal voluntary HIV testing and immediate ART	South Africa	General population (age not specified)	Not specified	42 years	Modelling	Cost-effectiveness analysis	Published literature/data	Modelling
#21 (Kasymova, Johns and Sharipova, 2009)	HIV testing and counselling/serial of HIV rapid test/not POC	Tajikistan	Youth population (aged 15–25 years)	Youth friendly health services	2 years	Cross-sectional study	Cost analysis	Primary data collection	Modelling
#22 (Kato *et al.*, 2013)	HIV testing type of test not specified/not POC, comparing: #1 Reference scenario #2 Targeted PTIT scenario #3 Universal PTIT scenario	Vietnam	Key population: PWID, MSM, FSWs, MCF, IDU and low-risk women (age not specified)	Health facility	50 years	Modelling	Cost effectiveness analysis	Published literature/data	Modelling
#23 (Kumar *et al.*, 2006)	HIV testing and counselling/rapid HIV test/not POC, comparing: #1 A universal testing #2 A targeted testing	India	Pregnant women (age not specified)	ANC clinic	Lifetime	Modelling	Cost–benefit analysis	Published literature/data	Modelling
#24 (Dandona *et al.*, 2009)	HIV voluntary counselling and testing/HIV rapid test/not POC	India	General population (age not specified)	VCT clinic	1 year	Cross-sectional study	Cost analysis	Primary data collection	Modelling
#25 (Mangenah *et al.*, 2019)	HIVST/HIV rapid testing/not POC	Malawi, Zambia, and Zimbabwe	General population (aged 15–59 years)	Community based distributing agent	1 year	Cross-sectional study	Cost analysis	Primary data collection	Econometric
#26 (McCreesh *et al.*, 2017)	HIV self-testing/HIV rapid test/not POC, comparing #1 Current coverage #2 Increased HIV testing (doubled)	Uganda	General population (aged under 51 years)	Door-to-door community-based	15 years	Modelling	Cost-effectiveness analysis	Published literature/data	Modelling
#27 (Minh *et al.*, 2012)	HIV voluntary counselling and testing/type of test not specified/not POC	Vietnam	General population (age not specified)	Facility based and freestanding VCT clinic	4 months	Costing study	Cost analysis	Primary data collection	Modelling
#28 (Sharma *et al.*, 2016)	HIV home-based partner education and testing (HOPE)/HIV rapid test/not POC, comparing: #1 Standard care (facility-based HIV testing) #2 Adding HOPE to standard care	Kenya	Partner of pregnant women (aged 0–59 years)	Home based	10 years	Modelling alongside randomized controlled trial	Cost-effectiveness analysis	Primary data collection	Modelling
#29 (Nelwan *et al.*, 2016)	HIV testing and counselling/HIV rapid test/not POC	Indonesia	Prisoner (age not specified)	Outpatient clinic in prison	3 years	Case–control study	Cost analysis	Primary data collection	Modelling
#30 (Luong Nguyen *et al.*, 2018)	HIV voluntary counselling and testing/HIV rapid test/not POC, comparing: #1 Current testing coverage of 62% #2 Scale up testing coverage to 90%	Kenya	General population (age not specified)	Health facility	20 years	Modelling	Cost-effectiveness analysis and budget impact analysis	Published literature/data	Modelling
#31 (Rely, 2003)	HIV counselling and testing/HIV rapid test/not POC, comparing: #1 Status quo (with 4% testing coverage) #2 Increase in coverage to 85%	Mexico	Pregnant women (age not specified)	Health facility	Lifetime	Modelling	Cost-effectiveness analysis	Published literature/data	Modelling
#32 (Tchuenche *et al.*, 2018)	VEID/DNA-PCR/not POC	Lesotho	HIV-exposed infants (0–2 weeks)	Health facility	1 year	Retrospective observational study	Cost analysis	Primary data collection	Modelling
#33 (Verstraaten *et al.*, 2017)	HIV voluntary counselling and testing/HIV rapid test/not POC	Indonesia	FSWs (age not specified)	Mobile VCT services	1 year		Cost analysis	Primary data collection	Modelling
#34 (Zang *et al.*, 2016)	HIV testing, comparing: #1 One4All (include testing, counselling, CD4 results, and viral load)/HIV rapid test/POC #2 Standard care/HIV rapid test+ western blot confirmatory test/POC	China	General population (aged 15–64 years old)	Hospital	1, 5 and 25 years	Modelling	Cost-effectiveness analysis alongside clustered randomized trial	Published literature/data	Modelling
#35 (Zhang *et al.*, 2015)	HIV testing/type of test not specified/not POC, comparing #1 Status quo #2 Reach universal coverage by 2015 #3 Reach universal coverage by 2017 #4 Reach universal coverage by 2022	Thailand	MSM (age not specified)	Health facility	3-,5-and 10-years	Modelling	Cost-effectiveness analysis	Primary data collection	Modelling

90–90–90 target: 90% of all people living with HIV will know their HIV status, 90% of all people with diagnosed HIV infection will receive sustained antiretroviral therapy and 90% of all people receiving antiretroviral therapy will have viral suppression. ANC, antenatal care; aPS, assisted partner service; ART, antiretroviral treatment; CD4, cluster of differentiation 4; DNA-PCR, deoxyribonucleic acid-polymerase chain reaction, a molecular diagnostic testing using DNA sequencing; HIVST, HIV self-testing; IDUs: injected drug users; MCF, male clients of female sex workers; POC: point-of-care; PTIT, HIV periodic testing and immediate treatment; PWID, people with injected drug; RPR, rapid plasma regain; VCT, HIV voluntary counselling and testing; VEID, very early infant diagnosis.

### Type of cost studies undertaken

Around half of the studies undertook a cost analysis (*N* = 17) and another half conducted a cost-effectiveness analysis (CEA) (*N* = 17) typically comparing facility-based HIV testing using rapid tests with laboratory-based testing, with two of those complemented their CEAs with budget impact analysis (BIA) (Cherutich *et al.*, 2018; Luong Nguyen *et al.*, 2018). One cost–benefit analysis of HIV testing in pregnant women was reported (Kumar *et al.*, 2006). Around half of the studies relied on primary data (*N* = 18) and the remaining half used a combination of published evidence and expert opinion (*N* = 17). Most studies took a modelling approach to the estimation of the costs of scaling up testing for HIV and/or syphilis (*N* = 25).

### Methods to assess cost according to scale

Of the 35 eligible studies, 10 measured how costs varied with scale (see Table 4). All other studies used a constant average cost assumption (i.e. no adjustment for scale) to estimate resource requirements of scaling up, implicitly assuming that costs were indifferent to scale and other potential cost drivers. Of the 10 studies measuring the impact of scale on costs, six conducted an econometric analysis by estimating the relationship between cost and cost determinants using regression analysis and remaining four are empirical studies (Forsythe, 2002; McConnel *et al.*, 2005; Dandona *et al.*, 2008a,b; Shelley *et al.*, 2015; Galárraga *et al.*, 2017; Mwenge *et al.*, 2017; Bautista-Arredondo *et al.*, 2018a; Mangenah *et al.*, 2019).

**Table 4:** Studies that report on how costs of testing change with scale

Ref	Definition of scaling up	Year (costs)	Units of output	Sample size	Timeframe for decision	Authors’ categorization of costs	Findings related to scale and cost	Results	Key drivers of costs identified^a^
#1 (Shelley *et al.*, 2015)	Increase in geographic coverage	2012	Number of facilities	Pilot: 5 facilities in two districts Scale up: 4 facilities in two districts	Short run	Incremental costs Start-up costs: personnel, per diems, conference hire, training equipment and supplies, and vehicle transport Capital costs: vehicles and computers Recurrent costs: personnel, supplies (syphilis testing, shared supplies, treatment), vehicle fuel and maintenance, quality assurance/control and supervision	Diseconomies of scale	Average unit cost per woman tested USD 3.19 (pilot) and USD 11.16 (scale up)	Geography and infrastructure: central level supervision and transport costs Managing the process of scaling up: quality assurance/control Other: higher RST kit cost and lower RST uptake
#3 (Bautista-Arredondo *et al.*, 2018a,b)	Increase in number of clients receiving a test	2013	Number of clients	414 540 clients in 141 HIV testing and counselling facility	Short run	Economic cost Personnel: staff salaries Recurrent inputs and services: HIV testing kit and treatment Capital: equipment (i.e. PCR machine, CD4 testing machine, refrigerator) and vehicles Training: Incl. opportunity costs of staff involved	Economies of scale	Average unit cost per client tested USD 30 Coefficient of scale^b^ = −0.44	Managing the process of scaling up: central-level financial decision, and task-shifting (i.e. reorganizing human resource to delegate some tasks to less specialized health workers) Fixed costs: the distribution of fixed cost to a larger number of people Other: services integration, external supervision, and different level of service delivery
#4 (Dandona *et al.*, 2008a,b)	Increase in number of clients receiving a test	2006	Number of clients	Pilot: 32 413 clients Scale up: 66 445 clients 17 hospital based VCT clinic	Short run	Economic cost Personnel: staff payroll Recurrent goods: HIV test kits, condoms, IEC (information, education, and communication) materials, medical supplies, and stationery Recurrent services: staff training, local travel, building maintenance and utilities Capital goods: furniture, medical and administrative equipment Building: based on area-specific rentals	Economies of scale	Average unit cost per client tested USD 5.46 (pilot) and USD 3.3 (scale up)	Fixed costs: the distribution of fixed cost to a larger number of people
#5 (Galárraga *et al.*, 2017)	Increase in number of clients receiving a test	2011	Number of clients per year	237 160 clients in 56 sites HTC clinic	Short run	Economic cost Personnel: staff and volunteer time Recurrent supplies: HIV test kits, condoms Recurrent operating costs: utilities and maintenance Capital goods: equipment—purchase, maintenance, and replacement Other inputs: administration, supervision, training	Economies of scale	Average cost per client tested is USD 7 Coefficient of scale^b^ = −0.18	Managing the process of scaling up: task shifting (i.e. using qualified lower level staff instead of physicians) Fixed costs: the distribution of fixed cost to a larger number of people
#8 (McConnel *et al.*, 2005)	Increase in number of clients receiving a test	2003	Number of clients	693 clients	Short run	Economic and financial costs Personnel: Staff and volunteer time Other recurrent goods and services: Staff and community training, campaign and publicity materials, utilities, stationary and donated HIV test kits, and condoms Capital: Office equipment and building mortgage	Economies of scale	Average cost per VCT client tested is USD 161.03 (pilot) and USD 53.02 (scale up)	Fixed costs: the distribution of fixed cost to a larger number of people
#13 (Mwenge *et al.*, 2017)	Increase in the total number of test kits distributed	2016	Number of test kits	A total of 7735 test kits distributed in 54 HIV testing services units	Short run	Economic and financial costs Capital costs: Buildings and storage, equipment, and vehicles Recurrent costs: Personnel, training, HIV testing commodities, general supplies, facility-level operation, and waste management Overhead costs: facility level and HTS centre-level	Economies of scale	Average cost per client tested is USD 4.92 (Malawi), USD 4.24 (Zambia) and USD 8.79 (Zimbabwe) Coefficient of scale^b^ = not provided	Fixed costs: the distribution of fixed cost to a larger number of people Personnel: staff salaries and training Other: service integration and lack of demand
#17 (Dandona *et al.*, 2008a,b)	Increase in number of clients receiving a test	2006	Number of clients	125 073 clients in 16 PMTCT centres	Short run	Economic and financial costs Rental: building and land Personnel: Staff time Capital goods: Furniture, medical and administrative equipment Recurrent goods: HIV test kits, Nevirapine, disposable supplies, stationary and miscellaneous item Recurrent services: Staff training, building maintenance and utilities, and waste disposal	Economies of scale	Average cost per client tested is USD 4.29 (pilot) and USD 1.61 (scale up)	Fixed costs: the distribution of fixed cost to a larger number of people
#18 (Dandona *et al.*, 2005)	Increase in number of clients receiving a test	2003	Number of clients	32 413 clients in 17 VCT clinics	Short run	Economic cost Salaries: staff and volunteer time Rentals: building and land Capital goods: Furniture and medical equipment Recurrent goods: HIV test kits, male condoms, IEC material, recurrent medical supplies, and stationery Recurrent services: Staff training, building maintenance and utilities, and waste disposal	Economies of scale	Average cost per client tested is USD 5.46 Coefficient of scale^b^ = −0.83	Fixed costs: the distribution of fixed cost to a larger number of people Other: lack of demand
#19 (Forsythe 2002)	Increase in number of clients receiving a test	1999	Number of clients	519 clients in three health centres	Short run	Economic costs Labour costs: staff salaries Materials and medication: HIV test kits, needles, syringes, and gloves Equipment and furniture: no detail provided Property and utilities: building rental value	Economies of scale	Average cost per client tested is USD 16 Coefficient of scale^b^ = not provided	Fixed costs: the distribution of fixed cost to a larger number of people
#25 (Mangenah *et al.*, 2019)	Increase in the total number of test kits distributed	2019	Number of test kits	A total of 349 719 test kits distributed in 71 sites	Short run	Economic and financial costs Start-up costs: training and community sensitization activities Capital costs: building and storage, equipment, and vehicle Recurrent costs: personnel, supplies, HIV self-test kits vehicle and building operation/maintenance, recurrent training, and waste management	Economies of scale	Average cost per kit distributed is USD 8.15 (Malawi), USD 16.42 (Zambia), and USD 13.84 (Zimbabwe) Coefficient of scale^b^ = not provided	Fixed costs: the distribution of fixed cost to a larger number of people

CD4, cluster of differentiation 4; DALY, Disability-adjusted life years; HTC, HIV testing and counselling; HTS, HIV testing services; PCR, polymerase chain reaction; PMTCT, prevention mother-to-child transmission; RST, Rapid syphilis testing; VCT, HIV voluntary counselling and testing.

a Categories for key drivers are summarized as geography and infrastructure, fixed costs, personnel, managing the process of scaling up and others, as discussed by Johns and Torres (2005).

b Coefficient of scale is a measure of association between average cost and level of scale.

### Impact of scale on costs

The six studies measuring the impact of scale on costs found that the average cost per person tested (most commonly for HIV using a rapid test) decreased as scale increased, demonstrating that economies of scale were possible (i.e. confirmed through a negative coefficient on the scale variable), with coefficient of scale is ranging from 0.18 to 0.83 (Dandona *et al.*, 2005; McConnel *et al.*, 2005; Galárraga *et al.*, 2017; Mwenge *et al.*, 2017; Bautista-Arredondo *et al.*, 2018b; Mangenah *et al.*, 2019). The main driver of these economies of scale is the distribution of fixed cost to a larger number of patients or outputs. The remaining four studies took an empirical-based approach whereby the relationship between scale and cost was based on observations of the actual cost at different levels of scale (Forsythe, 2002; McConnel *et al.*, 2005; Dandona *et al.*, 2008a,b; Shelley *et al.*, 2015). Three of the four studies showed that the costs of screening per person decreased as scale increased (Forsythe, 2002; McConnel *et al.*, 2005; Dandona *et al.*, 2008a,b). One empirical study reported an increase in the average cost per client tested as the intervention was rolled out to new sites, which was attributed to several factors including higher rapid point-of-care syphilis tests prices and lower rapid syphilis testing (RST) uptake in the targeted population (Shelley *et al.*, 2015).

### Fixed vs variable costs

All studies in this review were based on a short-run decision framework, during which the amount of fixed inputs could not be easily varied. The costs of scaling up HIV and syphilis testing tended to be narrowly defined as the cost of the test, personnel and associated consumables such as gloves and cotton swabs (*N* = 18). Only 10 out of 35 included studies separated the fixed and variable component of costs. A small number of studies (*N* = 8) attempted to include more cost items such as the costs of educational materials, monitoring and supervision or waste management. Few studies (*N* = 4) considered the costs of managing the scaling up process including the costs of quality management and investment in procuring new equipment. In addition to scale, other key drivers of cost included availability of transport infrastructure, variation in the price of local goods (e.g. test kits, medicines, fuel), costs and frequency of supervisory trips and the recruitment and training of health personnel, especially in remote areas.

### Quality of studies included

Table 5 summarizes the results of the appraisal. Most studies clearly reported the research question(s), perspective taken and the time horizon for the analysis (*N* = 29). No study justified their sample size for the costing and around half undertook a sensitivity analysis for major cost inputs (*N* = 19). There were widespread gaps in the methodological quality of estimating costs at scale. Specifically, less than half of the studies in this review estimated average costs at different levels of scale (*N* = 6), measured the relationship between average costs and scale (*N* = 9) or separated fixed and variable costs (*N* = 10), which are all necessary to accurately measure economies or diseconomies of scale (Kumaranayake, 2008).

**Table 5:** Results of the appraisal

References	Costs										Costs at scale			
	#1 Question(s)	#2 Perspective	#3 Time horizon	#4 Relevant inputs	#5 Methods for quantities	#6 Data source(s)	#7 Sample size	#8 Discount rate	#9 Sensitivity analysis	#10 Costs reporting	#11 Costs and scale	#12 Quantification	#13 Fixed and variable costs	#14 Factors other than scale

Shelley *et al.* (2015)	✓	✓	(✓)	✓	✓	X	(✓)	✓	✓	✓	(✓)	✓	✓	✓
Schackman *et al.* (2007)	✓	✓	X	(✓)	✓	✓	X	(✓)	✓	✓	(✓)	X	X	✓
Bautista-Arredondo *et al.* (2018a,b)	✓	✓	✓	✓	✓	✓	(✓)	(✓)	X	✓	✓	✓	✓	✓
Dandona *et al.* (2008a,b)	✓	(✓)	✓	✓	✓	✓	(✓)	✓	X	✓	(✓)	X	✓	X
Galárraga *et al.* (2017)	✓	X	✓	✓	✓	✓	(✓)	✓	X	✓	✓	✓	✓	✓
Hontelez *et al.* (2013)	✓	✓	✓	(✓)	(✓)	(✓)	(✓)	✓	✓	(✓)	(✓)	X	X	X
Ishikawa *et al.* (2016)	✓	✓	(✓)	(✓)	X	✓	X	(✓)	✓	(✓)	X	X	X	(✓)
McConnel *et al.* (2005)	✓	X	✓	✓	✓	✓	X	(✓)	X	✓	✓	X	✓	X
Ahaibwe G & Kasirye I (2013)	✓	X	✓	X	X	X	(✓)	(✓)	✓	(✓)	X	X	X	X
Alsallaq *et al.* (2017)	✓	✓	(✓)	(✓)	(✓)	✓	(✓)	(✓)	✓	✓	X	X	X	X
Cambiano *et al.* (2015)	✓	✓	(✓)	(✓)	(✓)	✓	X	(✓)	✓	(✓)	X	X	X	(✓)
Cherutich *et al.* (2018)	✓	✓	✓	✓	✓	✓	(✓)	(✓)	✓	✓	(✓)	X	(✓)	✓
Mwenge *et al.* (2017)	✓	✓	✓	✓	✓	✓	(✓)	√	✓	✓	✓	✓	✓	✓
Stuart *et al.* (2018)	✓	X	(✓)	(✓)	(✓)	(✓)	X	X	X	(✓)	(✓)	X	X	(✓)
Tromp *et al.* (2013)	✓	✓	✓	(✓)	✓	(✓)	(✓)	(✓)	√	(✓)	X	X	(✓)	✓
Zhuang *et al.* (2018)	✓	✓	✓	(✓)	(✓)	X	X	✓	X	(✓)	(✓)	X	X	X
Dandona *et al.* (2008a,b)	✓	(✓)	✓	✓	✓	✓	(✓)	✓	X	✓	✓	✓	✓	✓
Dandona *et al.* (2005)	✓	X	✓	✓	✓	✓	(✓)	✓	X	(✓)	(✓)	✓	✓	✓
Forsythe (2002)	✓	✓	✓	(✓)	✓	✓	X	(✓)	X	✓	X	✓	✓	✓
Granich *et al.* (2009)	✓	X	(✓)	(✓)	(✓)	(✓)	X	X	X	(✓)	X	X	X	X
Kasymova *et al.* (2009)	✓	✓	✓	✓	✓	✓	(✓)	✓	X	✓	(✓)	X	(✓)	X
Kato *et al.* (2013)	✓	X	✓	(✓)	X	✓	(✓)	(✓)	X	✓	(✓)	X	X	✓
Kumar *et al.* (2006)	✓	√	(✓)	(✓)	(✓)	(✓)	X	✓	✓	(✓)	(✓)	X	X	✓
Dandona *et al.* (2009)	✓	(✓)	✓	✓	✓	✓	(✓)	✓	✓	(✓)	(✓)	X	(✓)	✓
Mangenah *et al.* (2019)	✓	✓	✓	✓	✓	✓	(✓)	✓	✓	✓	✓	✓	✓	X
McCreesh *et al.* (2017)	✓	✓	✓	(✓)	X	✓	(✓)	(✓)	✓	(✓)	X	X	X	✓
Minh *et al.* (2012)	✓	✓	✓	(✓)	✓	✓	(✓)	✓	X	✓	X	X	(✓)	X
Sharma *et al.* (2016)	✓	✓	✓	✓	✓	(✓)	(✓)	✓	✓	✓	(✓)	X	(✓)	(✓)
Nelwan *et al.* (2016)	✓	✓	✓	(✓)	X	(✓)	X	X	X	(✓)	X	X	X	X
Luong Nguyen *et al.* (2018)	✓	(✓)	✓	✓	(✓)	✓	(✓)	✓	✓	✓	X	X	(✓)	X
Rely. (2003)	✓	✓	(✓)	(✓)	X	(✓)	X	(✓)	✓	(✓)	X	X	X	(✓)
Tchuenche *et al.* (2018)	✓	(✓)	✓	✓	✓	✓	(✓)	✓	(✓)	✓	X	X	X	✓
Verstraaten *et al.* (2017)	✓	✓	✓	✓	✓	(✓)	(✓)	(✓)	✓	✓	X	X	(✓)	X
Zang *et al.* (2016)	✓	✓	✓	(✓)	(✓)	✓	(✓)	✓	✓	(✓)	(✓)	X	X	(✓)
Zhang *et al.* (2015)	✓	(✓)	✓	✓	✓	✓	(✓)	(✓)	X	✓	(✓)	✓	(✓)	✓

✓: yes; X: no; (✓): partially addressed.

## Discussion

To expand access to HIV and syphilis testing and reach elimination targets, successful small-scale programmes need to cover broader populations in LMICs. The availability of reliable and detailed information on the resources required to do this is a key determinant of success (Johns and Torres, 2005; Kumaranayake, 2008; World Health Organization and ExpandNet, 2009; Mangham and Hanson, 2010). This review validates that scale is an important driver in determining the costs of HIV and syphilis testing programmes in resource-constrained health systems. It also reveals the potential for economies of scale (i.e. a reduction in average costs as the number of people tested increases) at least in the short run when structural changes to health systems (e.g. training, quality management and stock management) necessary for the large scale delivery of testing have not yet been undertaken.

Despite syphilis testing being widely recommended for use in LMICs, particularly in pregnancy (Newman *et al.*, 2013; Storey *et al.*, 2019), only two studies explored the costs of scaling up syphilis testing. Recent pilot studies have demonstrated the cost-effectiveness of HIV and syphilis screening using a dual rapid test over single HIV and syphilis tests or HIV testing alone (Bristow *et al.*, 2016). It has been argued that dual tests for HIV and syphilis may potentially contribute to economies of scale and scope (associate with the sharing of fixed costs across activities) in terms of start-up, training, quality management, supervision and monitoring while also serving to promote syphilis testing, which is lagging well behind HIV testing in many LMICs (Bristow *et al.*, 2016; Taylor *et al.*, 2017). While there were many studies that found community-based strategies are effective in increasing uptake of testing (Asiimwe *et al.*, 2017; Ahmed *et al.*, 2018), this review revealed a lack of attention paid to the economic impact of scaling up these strategies. Many key populations have expressed a strong preference for community-based testing, which is seen as less stigmatizing and more accessible (Suthar *et al.*, 2013). Despite the growing importance of the private sector in the delivery of HIV and syphilis care in LMICs (Rao *et al.*, 2011; Wang *et al.*, 2017), only one study exploring the costs of scaling up the delivery of testing through private or non-government providers (Verstraaten *et al.*, 2017) was identified. Further research on the costs of scaling up syphilis testing and modelling community-based HIV and syphilis testing in public and private sectors are needed.

Our review highlighted that while the overall standard of reporting costs was reasonable, with most studies partially or fully addressing seven or more of the ten questions, shortcomings manifested in two key areas. First, none of the studies in this review justified their sample size for the costing. Economic evaluations often require larger sample sizes for adequate power compared to a typical clinical study evaluating the health impact of a testing intervention (Taylor *et al.*, 2017). However, clinical studies and economic evaluations generally investigate differences in outcomes at the individual level. Cost drivers, such as scale, are better identified through multi-site cost studies with the production unit or clinic as the unit of analysis (Tagar *et al.*, 2014). A representative sample should capture differences in clinic characteristics such as geographical setting, ownership, and management systems. However, commonly the trial sample size or convenience sampling, in a limited number of sites, determines sample size for a full or partial economic evaluation. Given the considerable uncertainties around the costs of going to scale including the varying cost of procuring and delivering test kits (Johns and Torres, 2005; Shelley *et al.*, 2015), it was surprising that almost half of the studies failed to explore the influence of different key inputs on unit costs or total programme costs by conducting sensitivity analysis. For example, volume purchasing, which is being explored in many LMICs, is likely to impact the cost and uptake of rapid diagnostic tests (Taylor *et al.*, 2017) and worth closer consideration.

From a methodological viewpoint, the appraisal revealed that only eight studies adjusted for changes in unit costs as HIV and syphilis testing was scaled up (McConnel *et al.*, 2005; Dandona *et al.*, 2008a,b; Shelley *et al.*, 2015; Galárraga *et al.*, 2017; Mwenge *et al.*, 2017; Bautista-Arredondo *et al.*, 2018a; Mangenah *et al.*, 2019). Scaling up HIV and syphilis testing programmes typically involves transporting supplies longer distances and to more remote areas compared to pilot programs, which can lead to variations in the prices of consumables such as testing kits. The impact on programme outcomes must also be considered alongside any changes in costs. Sweeney *et al.* (2014) have argued that, while devolving supervision and monitoring of RST in Tanzania to authorities at the sub-national level may lead to reductions in the frequency and cost of external quality assurance, this may pose challenges for quality maintenance (Sweeney et al., 2014) . Our appraisal also revealed that despite majority (22 out of 35) of studies have clearly stated their perspective, only three of these adopted a societal perspective. This means that a significant proportion of direct and indirect cost incurred by patients for accessing tests is not considered. Moreover, while most studies included a range of recurrent costs (e.g. staff time, training, testing commodities and other medical supplies) and capital costs (e.g. vehicles and computers) and were focused at the level of a clinic or health centre, few acknowledged the required investments in infrastructure (e.g. quality management, reporting system) and broader health systems strengthening needed as HIV and syphilis testing programmes are scaled up to the national level across all clinics and facilities (Kumaranayake *et al.*, 2001). These ‘higher level’ investments refer ‘to the policy, political, legal, regulatory, budgetary or other health systems changes needed to institutionalize new innovations at the national or sub-national level’ (World Health Organization and ExpandNet, 2010).

Another area for methodological improvement relates to the quantification of the relationship between average costs and the scale of delivering HIV or syphilis testing. Most studies undertook a simple form of modelling whereby costs were scaled up linearly. For these studies, an empirical average cost associated with a testing programme was multiplied by a factor representing activity at a larger scale. For example, if the unit cost per person tested for HIV and syphilis is United States Dollar (USD) 10 for 100 people then expanding coverage by another 50 people would be USD 500 (Kumaranayake, 2008). Only 10 of the 35 studies developed non-linear cost functions, allowing some costs to be fixed regardless of the size of the population reached—e.g. medical equipment, vehicles or buildings—leading to economies of scale. For one study on the roll out of syphilis rapid testing in Zambia, lower clinic catchment populations combined with higher unit costs for transport, supervision and test kits reduced the economies of scale achieved in the high coverage pilot sites (Shelley *et al.*, 2015). This resonates with economic theory as output increases average costs will first fall and then rise, following a ‘u’-shaped curve (Guinness, Kumaranayake and Hanson, 2007; Kumaranayake, 2008). This small subset of studies represents an important step forward in providing a more accurate estimation of the costs of scaling up HIV and syphilis testing interventions in LMICs, painting a more complex picture of the relationship between scale and costs.

There are some limitations of this review that need to be acknowledged. Studies were restricted to those found in the published literature, a potential source of reporting bias. In addition, the studies identified varied by analytical approach (empirical, econometric or model), testing intervention and types of costs measured. This diversity prevented the pooling of results for a meta-analysis. Instead, studies were qualitatively reviewed, and their results and characteristics tabulated which helped to highlight evidence gaps and methodological weaknesses.

In summary, this review highlights evidence of the relationship between the costs and scale of delivering HIV and syphilis testing in LMICs. What is less clear is how costs change with scale and in turn the potential for economies of scale. Scaling up costs linearly, an assumption that underpins most studies in this review, and runs the risk of misleading policymakers as to the true costs of providing universal access to HIV and syphilis testing. Collecting empirical cost data alongside the roll-out of HIV and syphilis testing is a priority. Financing and budgeting for the scale up of HIV and syphilis testing will benefit from the monitoring of costs across different sites, contexts and settings as well as over time, for greater consistency in the categorization of fixed and variables costs, and the inclusion of costs associated with strengthening health systems to support quality assurance and stock management systems.

## Supplementary data

Supplementary data are available at *Health Policy and Planning* online

## Supplementary Material

czab030_Supp

## References

[czab030-B1] Ahaibwe G , Kasirye I. 2013. HIV/AIDS prevention interventions in Uganda: a policy simulation. Research Series 167527, Economic Policy Research Centre (EPRC).

[czab030-B2] Ahmed S , Autrey J, Katz IT et al 2018. Why do people living with HIV not initiate treatment? A systematic review of qualitative evidence from low- and middle-income countries. Social Science & Medicine 213: 72–84. 30059900 10.1016/j.socscimed.2018.05.048 PMC6813776

[czab030-B3] Alsallaq RA , Buttolph J, Cleland CM et al 2017. The potential impact and cost of focusing HIV prevention on young women and men: a modeling analysis in western Kenya. PLoS One 12: e0175447. 28403211 10.1371/journal.pone.0175447 PMC5389814

[czab030-B4] Asiimwe S , Ross JM, Arinaitwe A et al 2017. Expanding HIV testing and linkage to care in southwestern Uganda with community health extension workers. Journal of the International AIDS Society 20(Suppl 4): 21633. 28770598 10.7448/IAS.20.5.21633 PMC5577731

[czab030-B5] Bautista-Arredondo S , La Hera-Fuentes G, Contreras-Loya D et al 2018a. Efficiency of HIV services in Nigeria: determinants of unit cost variation of HIV counseling and testing and prevention of mother-to-child transmission interventions. PLoS One 13(9): e0201706. 30192765 10.1371/journal.pone.0201706 PMC6128456

[czab030-B6] Bautista-Arredondo S , Colchero MA, Amanze OO et al 2018b. Explaining the heterogeneity in average costs per HIV/AIDS patient in Nigeria: the role of supply-side and service delivery characteristics. PLoS One 13(5): e0194305. 29718906 10.1371/journal.pone.0194305 PMC5931468

[czab030-B7] Bert F , Gualano MR, Biancone P et al 2018. HIV screening in pregnant women: a systematic review of cost-effectiveness studies. The International Journal of Health Planning and Management 33: 31–50. 28556453 10.1002/hpm.2418

[czab030-B8] Bristow CC , Larson E, Anderson LJ et al 2016. Cost-effectiveness of HIV and syphilis antenatal screening: a modelling study. Sexually Transmitted Infections 92: 340–6. 26920867 10.1136/sextrans-2015-052367 PMC4956558

[czab030-B9] Cambiano V , Ford D, Mabugu T et al 2015. Assessment of the potential impact and cost-effectiveness of self-testing for HIV in low-income countries. Journal of Infectious Diseases 212: 570–7. 10.1093/infdis/jiv040 PMC4512606 25767214

[czab030-B10] Cherutich P , Farquhar C, Wamuti B et al; for the aPS Study Group. 2018. HIV partner services in Kenya: a cost and budget impact analysis study. BMC Health Services Research 18: 721. 30223833 10.1186/s12913-018-3530-y PMC6142360

[czab030-B11] Dandona L , Sisodia P, Ramesh YK et al 2005. Cost and efficiency of HIV voluntary counselling and testing centres in Andhra Pradesh, India. The National Medical Journal of India 18: 26–31. 15835489

[czab030-B12] Dandona L , Kumar SP, Ramesh YK et al 2008a. Changing cost of HIV interventions in the context of scaling-up in India. AIDS 22: S43–9. 18664952 10.1097/01.aids.0000327622.24232.aa PMC3688470

[czab030-B13] Dandona L , Kumar SP, Ramesh YK et al 2008b. Outputs, cost and efficiency of public sector centres for prevention of mother to child transmission of HIV in Andhra Pradesh, India. BMC Health Services Research 8: 26. 18234117 10.1186/1472-6963-8-26 PMC2267788

[czab030-B14] Dandona L Kumar SP Kumar GA et al. Economic analysis of HIV prevention interventions in Andhra Pradesh state of India to inform resource allocation. *AIDS* 23: 233–42. 10.1097/QAD.0b013e328320accc 19112690

[czab030-B15] Fleming K A Naidoo M Wilson M et al. 2017. An Essential Pathology Package for Low- and Middle-Income Countries. *American Journal of Clinical Pathology* 147: 15–32. 10.1093/ajcp/aqw143 28158414 28158414

[czab030-B16] Forsythe S. 2002. Assessing the cost and willingness to pay for voluntary HIV counselling and testing in Kenya. Health Policy and Planning 17: 187–95. 12000779 10.1093/heapol/17.2.187

[czab030-B17] Galárraga O , Wamai RG, Sosa-Rubí SG et al 2017. HIV prevention costs and their predictors: evidence from the ORPHEA Project in Kenya. Health Policy and Planning 32: 1407–16. 29029086 10.1093/heapol/czx121 PMC5886164

[czab030-B18] Getzen TE. 2014. *Health Economics and Financing*, 5th edn, 3–6. https://www.wiley.com/en-us/Health+Economics+and+Financing%2C+5th+Edition-p-9781118184905, accessed 28 July 2020.

[czab030-B19] Gomez GB , Mudzengi DL, Bozzani F et al 2020. Estimating cost functions for resource allocation using transmission models: a case study of tuberculosis case finding in South Africa. Value in Health 23: 1606–12. 33248516 10.1016/j.jval.2020.08.2096

[czab030-B20] Granich RM , Gilks CF, Dye C et al 2009. Universal voluntary HIV testing with immediate antiretroviral therapy as a strategy for elimination of HIV transmission: a mathematical model. The Lancet 373: 48–57. 10.1016/S0140-6736(08)61697-9 19038438

[czab030-B21] Guinness L , Kumaranayake L, Hanson K. 2007. A cost function for HIV prevention services: is there a “u”—shape?, Cost Effectiveness and Resource Allocation 5: 13. 17983475 10.1186/1478-7547-5-13 PMC2206005

[czab030-B22] Hontelez JAC , Lurie MN, Bärnighausen T et al 2013. Elimination of HIV in South Africa through expanded access to antiretroviral therapy: a model comparison study. PLoS Medicine 10: e1001534. 24167449 10.1371/journal.pmed.1001534 PMC3805487

[czab030-B23] Hook EW. 2017. Syphilis. The Lancet 389: 1550–7. 10.1016/S0140-6736(16)32411-4 27993382

[czab030-B24] Husereau D , Drummond M, Petrou S et al 2013. Consolidated Health Economic Evaluation Reporting Standards (CHEERS) statement. The European Journal of Health Economics 14: 367–72. 23526140 10.1007/s10198-013-0471-6

[czab030-B25] Ishikawa N , Dalal S, Johnson C et al 2016. Should HIV testing for all pregnant women continue? Costeffectiveness of universal antenatal testing compared to focused approaches across high to very low HIV prevalence settings. Journal of the International AIDS Society, 19: 21212. 27978939 10.7448/IAS.19.1.21212 PMC5159683

[czab030-B26] Johns B. 2015. The link between modelling and doing. The Lancet HIV 2: e174–e175. 26422998 10.1016/S2352-3018(15)00036-3

[czab030-B27] Johns B , Baltussen R. 2004. Accounting for the cost of scaling-up health interventions. Health Economics 13: 1117–24. 15386683 10.1002/hec.880

[czab030-B28] Johns B , Torres TT. 2005. Costs of scaling up health interventions: a systematic review. Health Policy and Planning, 20: 1–13. 15689425 10.1093/heapol/czi001

[czab030-B29] Joint United Nations Programme on HIV/AIDS. 2018. *UNAIDS* Data 2018. https://www.unaids.org/sites/default/files/media_asset/unaids-data-2018_en.pdf, accessed 10 September 2018.

[czab030-B30] Kamb ML , Newman LM, Riley PL et al 2010. A road map for the global elimination of congenital syphilis. Obstetrics and Gynecology International 2010: 1–6. 10.1155/2010/312798 PMC2913802 20706693

[czab030-B31] Kasymova N , Johns B, Sharipova B. 2009. The costs of a sexually transmitted infection outreach and treatment programme targeting most at risk youth in Tajikistan. Cost Effectiveness and Resource Allocation 7: 19. 19883515 10.1186/1478-7547-7-19 PMC2780377

[czab030-B32] Kato M , Granich R, Bui DD et al 2013. The potential impact of expanding antiretroviral therapy and combination prevention in Vietnam: towards elimination of HIV transmission. Journal of Acquired Immune Deficiency Syndromes 63(5):142–9. doi:10.1097/QAI.0b013e31829b535b. 23714739 10.1097/QAI.0b013e31829b535b PMC3814627

[czab030-B33] Kumar M , Birch S, Maturana A et al 2006. Economic evaluation of HIV screening in pregnant women attending antenatal clinics in India. Health Policy 77: 233–43. 16126300 10.1016/j.healthpol.2005.07.014

[czab030-B34] Kumaranayake L. 2008. The economics of scaling up: cost estimation for HIV/AIDS interventions. AIDS 22: S23–S33. 18664950 10.1097/01.aids.0000327620.47103.1d

[czab030-B35] Kumaranayake L , Kurowski C, Conteh L. 2001. *Costs of Scaling Up Priority Health Interventions in Low-Income and Selected Middle-Income Countries: Methodology and Estimates*. https://www.researchgate.net/publication/237799921_Costs_of_scaling_up_Priority_Health_Interventions_in_Low-income_and_selected_Middle-income_Countries_Methodology_and_Estimates, accessed 28 July 2020.

[czab030-B36] Luong Nguyen LB , Yazdanpanah Y, Maman D et al 2018. Voluntary community human immunodeficiency virus testing, linkage, and retention in care interventions in Kenya: modeling the clinical impact and cost-effectiveness. Clinical Infectious Diseases 67: 719–26. 29746619 10.1093/cid/ciy173 PMC6094004

[czab030-B37] Mabey DC , Sollis KA, Kelly HA et al 2012. Point-of-care tests to strengthen health systems and save newborn lives: the case of syphilis. PLoS Med 9: e1001233. 22719229 10.1371/journal.pmed.1001233 PMC3373627

[czab030-B38] Mangenah C , Mwenge L, Sande L et al 2019. Economic cost analysis of door-to-door community-based distribution of HIV self-test kits in Malawi, Zambia and Zimbabwe. Journal of the International AIDS Society 22: e25255. 30907499 10.1002/jia2.25255 PMC6432106

[czab030-B39] Mangham LJ , Hanson K. 2010. Scaling up in international health: what are the key issues? Health Policy and Planning 25: 85–96. 20071454 10.1093/heapol/czp066

[czab030-B40] McConnel CE , Stanley N, Du Plessis J-A et al 2005. The cost of a rapid-test VCT clinic in South Africa. South African Medical Journal 95: 968–71. 16465359

[czab030-B41] McCreesh N , Andrianakis I, Nsubuga RN et al 2017. Universal test, treat, and keep: improving ART retention is key in cost-effective HIV control in Uganda. BMC Infectious Diseases 17: 322. 28468605 10.1186/s12879-017-2420-y PMC5415795

[czab030-B42] McHugh ML. 2012. Interrater reliability: the kappa statistic. Biochemia Medica, 22: 276–82. 23092060 PMC3900052

[czab030-B43] Mikkelsen E , Hontelez JAC, Jansen MPM et al 2017. Evidence for scaling up HIV treatment in Sub-Saharan Africa: a call for incorporating health system constraints. PLoS Medicine 14: e1002240. 28222126 10.1371/journal.pmed.1002240 PMC5319640

[czab030-B44] Minh HV , Bach TX, Mai NY et al 2012. The cost of providing HIV/AIDS counseling and testing services in Vietnam. Value in Health Regional Issues 1: 36–40. 29702824 10.1016/j.vhri.2012.03.012

[czab030-B45] Moher D , Liberati A, Tetzlaff J et al; The PRISMA Group 2009. Preferred reporting items for systematic reviews and meta-analyses: the PRISMA statement. PLoS Medicine 6: e1000097. 19621072 10.1371/journal.pmed.1000097 PMC2707599

[czab030-B46] Mwenge L , Sande L, Mangenah C et al 2017. Costs of facility-based HIV testing in Malawi, Zambia and Zimbabwe. PLoS One 12: e0185740. 29036171 10.1371/journal.pone.0185740 PMC5642898

[czab030-B47] Nelwan EJ , Isa A, Alisjahbana B et al 2016. Routine or targeted HIV screening of Indonesian prisoners. International Journal of Prisoner Health 12: 17–26. 26933989 10.1108/IJPH-04-2015-0012

[czab030-B48] Newman L , Kamb M, Hawkes S et al 2013. global estimates of syphilis in pregnancy and associated adverse outcomes: analysis of multinational antenatal surveillance data. PLoS Medicine 10: e1001396. 23468598 10.1371/journal.pmed.1001396 PMC3582608

[czab030-B49] Peacock SJ , Richardson JRJ, Carter R et al 2007. Priority setting in health care using multi-attribute utility theory and programme budgeting and marginal analysis (PBMA). Social Science & Medicine 64: 897–910. 17141930 10.1016/j.socscimed.2006.09.029

[czab030-B50] Rao P Gabre-Kidan T Mubangizi D B et al. 2011. Leveraging the private health sector to enhance HIV service delivery in lower-income countries. *Journal of Acquired Immune Deficiency Syndromes* 57 (Suppl 2): S116–9. 10.1097/QAI.0b013e31821ed719 21857294 21857294

[czab030-B51] Rely K. 2003. Cost-effectiveness of strategies to reduce mother-to-child HIV transmission in Mexico, a low-prevalence setting. Health Policy and Planning 18: 290–8. 12917270 10.1093/heapol/czg035

[czab030-B52] Ryan R ; Cochrane Consumer and Communication Review Group. 2013. Cochrane Consumers and Communication Review Group: data synthesis and analysis. http:\\cccrg.cochrane.org, June 2013, accessed 28 July 2020.

[czab030-B53] Sanders GD , Bayoumi AM, Sundaram V et al 2005. Cost-effectiveness of screening for HIV in the era of highly active antiretroviral therapy. New England Journal of Medicine 352: 570–85. 10.1056/NEJMsa042657 15703422

[czab030-B54] Schackman BR , Neukermans CP, Fontain SNN et al 2007. Cost-effectiveness of rapid syphilis screening in Prenatal HIV testing programs in Haiti. PLoS Medicine 4: e183. 17535105 10.1371/journal.pmed.0040183 PMC1880854

[czab030-B55] Sharma M , Farquhar C, Ying R et al 2016. Modeling the cost-effectiveness of home-based HIV testing and education (HOPE) for pregnant women and their male partners in Nyanza Province, Kenya. Journal of Acquired Immune Deficiency Syndromes 72: S174–S180. 27355506 10.1097/QAI.0000000000001057 PMC5113236

[czab030-B56] Shelley KD , Ansbro ÉM, Ncube AT et al 2015. Scaling down to scale up: a health economic analysis of integrating point-of-care syphilis testing into antenatal care in Zambia during pilot and national rollout implementation. PLoS One 10: e0125675. 25970443 10.1371/journal.pone.0125675 PMC4430530

[czab030-B57] Simmons R , Shiffman J. 2007. '*Scaling Up Health Service Innovations: A Framework for Action', in Scaling up health service delivery: from pilot innovations to policies and programmes, pp 1-30*. https://www.who.int/immunization/hpv/deliver/scalingup_health_service_delivery_who_2007.pdf, accessed 24 May 2018.

[czab030-B58] Storey A , Seghers F, Pyne-Mercier L et al 2019. Syphilis diagnosis and treatment during antenatal care: the potential catalytic impact of the dual HIV and syphilis rapid diagnostic test. The Lancet Global Health 7: e1006–e1008. 31303285 10.1016/S2214-109X(19)30248-7 PMC6759458

[czab030-B59] Stuart RM , Fraser-Hurt N, Kerr CC et al 2018. The City of Johannesburg can end AIDS by 2030: modelling the impact of achieving the Fast-Track targets and what it will take to get there: modelling. Journal of the International AIDS Society 21: e25068. 10.1002/jia2.25068 PMC5810342 29359533

[czab030-B60] Suthar AB , Ford N, Bachanas PJ et al 2013. Towards universal voluntary HIV testing and counselling: a systematic review and meta-analysis of community-based approaches. PLoS Medicine 10: e1001496. 23966838 10.1371/journal.pmed.1001496 PMC3742447

[czab030-B0301563] Sweeney S , Mosha J F, Terris-Prestholt F et al 2014. The costs of accessible quality assured syphilis diagnostics: informing quality systems for rapid syphilis tests in a Tanzanian setting. *Health Policy and Planning* 29: 633–641. 10.1093/heapol/czt049 23894075

[czab030-B61] Tagar E , Sundaram M, Condliffe K et al 2014. Multi-Country analysis of treatment costs for HIV/AIDS (match): facility-level art unit cost analysis in Ethiopia, Malawi, Rwanda, South Africa and Zambia. PLoS One 9: e108304. 25389777 10.1371/journal.pone.0108304 PMC4229087

[czab030-B62] Taylor MM , Peeling RW, Toskin I et al 2017. Role of dual HIV/syphilis test kits in expanding syphilis screening. Sexually Transmitted Infections 93: 458–9. 28778981 10.1136/sextrans-2017-053301 PMC6748393

[czab030-B63] Tchuenche M , Gill MM, Bollinger L et al 2018. Estimating the cost of diagnosing HIV at birth in Lesotho. PLoS One 13: e0202420. 30110377 10.1371/journal.pone.0202420 PMC6093690

[czab030-B64] Tromp N , Siregar A, Leuwol B et al 2013. Cost-effectiveness of scaling up voluntary counselling and testing in West-Java, Indonesia. Acta Medica Indonesiana 45: 17–25. 23585404

[czab030-B65] UNICEF. 2020. *Elimination of Mother-to-Child Transmission*. https://data.unicef.org/topic/hivaids/emtct/, accessed 20 November 2020.

[czab030-B66] UNICEF East Asia and Pacific Regional Office.2016. *Progress Review and Road Map: Elimination of Parent-to-Child Transmission of HIV and Syphilis in Asia and the Pacific in 2015 and Beyond*. http://www.wpro.who.int/hiv/documents/topics/pmtct/20160920-eptct-progress-report/en/, accessed 10 May 2015.

[czab030-B67] Vassall A , Sweeney S, Kahn J et al 2017. *Reference Case for Estimating the Costs of Global Health Services and Interventions*. https://researchonline.lshtm.ac.uk/id/eprint/4653001.

[czab030-B68] Verstraaten EJM , Beeren FMM, Janssen JLC et al 2017. Comparative cost analysis of four interventions to prevent HIV transmission in Bandung, Indonesia. Acta Medica Indonesiana 49: 236–42. 29093234

[czab030-B69] Vita MG. 1990. Exploring hospital production relationships with flexible functional forms. Journal of Health Economics 9: 1–21. 10105280 10.1016/0167-6296(90)90038-5

[czab030-B70] Wang W , Temsah G, Mallick L. 2017. The impact of health insurance on maternal health care utilization: evidence from Ghana, Indonesia and Rwanda. Health Policy and Planning 32: 366–75. 28365754 10.1093/heapol/czw135 PMC5400062

[czab030-B71] Wiseman V , Mitton C, Doyle-Waters MM et al 2016. Using economic evidence to set healthcare priorities in low-income and lower-middle-income countries: a systematic review of methodological frameworks. Health Economics 25: 140–61. 26804361 10.1002/hec.3299 PMC5066677

[czab030-B72] World Bank.2019. *World Bank Country and Lending Groups – World Bank Data Help Desk*. The World Bank. https://datahelpdesk.worldbank.org/knowledgebase/articles/906519-world-bank-country-and-lending-groups, accessed 3 June 2019.

[czab030-B73] World Health Organization. 2016. *Global Health Sector Strategy on HIV 2016–2021*. https://apps.who.int/iris/bitstream/handle/10665/246178/WHO-HIV-2016.05-eng.pdf%253Bjsessionid=AA9223172C786025B9C3CE6274434C7A?sequence=1, accessed 27 December 2020.

[czab030-B74] World Health Organization.2017. *Global Guidance on Criteria and Processes for validation: Elimination of Mother-to-Child Transmission of HIV and Syphilis, Second Edition*. https://apps.who.int/iris/bitstream/handle/10665/259517/9789241513272-eng.pdf;jsessionid=33560A2E3646421ECF49F5C8FD063D3B?sequence=1, accessed 10 January 2020.

[czab030-B76] World Health Organization.2018. *2018 Report on global sexually transmitted infection surveillance*, *WHO Library Cataloguing*. http://apps.who.int/bookorders, accessed 22 November 2019.

[czab030-B77] World Health Organization and ExpandNet.2009. *Practical Guidance for Scaling Up Health Service Innovations*. Geneva, Switzerland: World Health Organization. ISBN: 9789241598521.

[czab030-B78] World Health Organization and ExpandNet.2010. *Nine Steps for Developing a Scaling-Up Strategy*. Geneva, Switzerland: World Health Organization.

[czab030-B79] Zang X , Tang H, Min JE et al 2016. Cost-effectiveness of the “One4All” HIV linkage intervention in Guangxi Zhuang Autonomous Region, China. PLoS One 11: e0167308. 27893864 10.1371/journal.pone.0167308 PMC5125690

[czab030-B80] Zhang L , Phanuphak N, Henderson K et al 2015. Scaling up of HIV treatment for men who have sex with men in Bangkok: a modelling and costing study. The Lancet HIV, 2: e200–e207. 26423002 10.1016/S2352-3018(15)00020-X

[czab030-B81] Zhuang X , Peng P, Sun H et al 2018. Scaling up human immunodeficiency virus screening and antiretroviral therapy among men who have sex with men to achieve the 90-90-90 targets in China. Sexually Transmitted Diseases 45: 343–9. 29465693 10.1097/OLQ.0000000000000744

