# Supplementary material for: The costs of scaling up HIV and syphilis testing in low- and middle-income countries: a systematic review
Source: Health Policy Plan. 2021 Mar 9;36(6):939–54. doi: 10.1093/heapol/czab030 (PMC8227996; doi:10.1093/heapol/czab030)
Supplement: czab030_Supp [file czab030_supp.zip › Supplementary file.docx]

**Appendix 1.** Searching strategies used for Medline via Ovid

| **Searching strategies for Medline via Ovid** | |
| --- | --- |
| Condition (infectious disease) | Exp HIV/ OR exp HIV infections/ OR exp Acquired Immunodeficiency Syndrome/ OR exp HIV antibodies/ OR HIV antigens/ OR HIV.mp. OR AIDS.mp. OR exp Syphilis/ OR exp Syphilis Congenital/ OR syphilis.m.p. |
| **AND** | |
| Intervention (testing OR screening) | Exp mass screening/ OR exp Diagnostic Tests, Routine/ OR exp Point-of-Care Testing/ OR exp Prenatal Diagnosis/ OR exp Syphilis Serodiagnosis/ OR (screen* adj15 (HIV OR Syphilis OR AIDS)).mp. OR (test* adj15 (HIV OR Syphilis OR AIDS)).mp. |
| **AND** | |
| Outcome (cost OR economic) | Exp “Costs and Cost Analysis”/ exp Resource Allocation/ OR cost.mp. OR costs.mp. OR costing.mp. |
| **AND** | |
| Countries (LMICs)^[[1]](#footnote-1)^ | Developing Countries.sh,kf. OR (Africa or Asia or Caribbean or West Indies or South America or Latin America or Central America).hw,kf,ti,ab,cp. OR ((developing or less* developed or under developed or underdeveloped or middle income or low* icome or underserved or underserved or deprived or poor*) adj (countr* or nation? or population? or world)).ti,ab. OR ((developing or less* developed or under developed or underdeveloped or middle income or low* income) adj (economy or economies)).ti,ab. OR (low* adj (gdp or gnp or gross domestic or gross national)).ti,ab. OR (low adj3 middle adj3 countr*).ti,ab. OR (lmic or lmics or third world or lami countr*).ti,ab. OR Afghanistan; bangladesh/ or bhutan/ or india/ or nepal/ or pakistan/ or sri lanka/or angola/ or benin/ or botswana/ or burkina faso/ or burundi/ or cameroon/ or cape verde/ or central african republic/ or chad/ or comoros/ or congo/ or cote d'ivoire/ or democratic republic congo/ or djibouti/ or equatorial guinea/ or eritrea/ or ethiopia/ or gabon/ or gambia/ or ghana/ or guinea/ or guinea-bissau/ or kenya/ or lesotho/ or liberia/ or madagascar/ or malawi/ or mali/ or mozambique/ or namibia/ or niger/ or nigeria/ or rwanda/ or senegal/ or sierra leone/ or somalia/ or south africa/ or south sudan/ or sudan/ or swaziland/ or tanzania/ or togo/ or uganda/ or zambia/ or zimbabwe/ cuba/ or dominica/ or dominican republic/ or grenada/ or haiti/ or jamaica/ or saint lucia/ or "saint vincent and the grenadines"/ iran/ or iraq/ or jordan/ or lebanon/ or syrian arab republic/ or "turkey (republic)"/ or yemen/armenia/ or azerbaijan/ or belarus/ or "georgia (republic)"/ or kazakhstan/ or kyrgyzstan/ or moldova/ or russian federation/ or tajikistan/ or turkmenistan/ or ukraine/ or uzbekistan/ cambodia/ or indonesia/ or laos/ or malaysia/ or myanmar/ or papua new guinea/ or thailand/ or timor-leste/ or vietnam/ algeria/ or egypt/ or libyan arab jamahiriya/ or mauritania/ or morocco/ or tunisia/ belize/ or costa rica/ or el salvador/ or guatemala/ or honduras/ or nicaragua/ far east/ or china/ or mongolia/ or philippines/ south america/ or bolivia/ or brazil/ or colombia/ or ecuador/ or guyana/ or paraguay/ or peru/ or suriname/ or venezuela/ "federated states of micronesia"/ or fiji/ or kiribati/ or marshall islands/ or nauru/ or papua new guinea/ or samoan islands/ or solomon islands/ or timor-leste/ or tonga/ or tuvalu/ or vanuatu/ "sao tome and principe"/ maldives/ or mauritius/ albania/ or "bosnia and herzegovina"/ or bulgaria/ or kosovo/ or "macedonia (republic)"/ or "montenegro (republic)"/ or russian federation/ or serbia/ Mexico/; “west bank and gaza”.hw,kf,ti,ab,cp. |

1. Constructed based on the modification of Cochrane LMICs Filter(Cochrane, 2012) that customised to the World Bank Country and Lending Group classification in the current 2019 fiscal year(World Bank, 2019). [↑](#footnote-ref-1)
