# Supplementary material for: The costs of scaling up HIV and syphilis testing in low- and middle-income countries: a systematic review
Source: Health Policy Plan. 2021 Mar 9;36(6):939–54. doi: 10.1093/heapol/czab030 (PMC8227996; doi:10.1093/heapol/czab030)
Supplement: czab030_Supp [file czab030_supp.zip › Table 1.docx]

Table 1. List of data extraction variables

| **Table 1.** Data extraction variables | |
| --- | --- |
| **Study characteristics** | |
| Intervention(s) | Type of HIV and/or syphilis testing activity or programme |
| Country | Location of study |
| Study population | Group targeted by intervention(s) |
| Setting | Service through which the intervention is delivered (e.g. health centre, hospital, etc.) and sector (e.g. public/private) |
| Time horizon | The duration over which costs and/or consequences are calculated |
| Study design | Randomised controlled trial, cross-sectional, cohort, case-control, modelling |
| Type of economic analysis (and ratio if applicable) | Cost analysis, cost‐effectiveness, cost‐utility, or cost–benefit analysis. Includes ratio used (e.g. cost per DALY averted) |
| Data source(s) | Primary data collection, expert/stakeholder opinion, published data or literature or combination of those |
| Analytical approach to measure costs at scale | Econometric, empirical, modelling or a hybrid of these approaches (Kumaranayake, 2008) |
| **Costs of scaling-up** | |
| Definition of scaling up | As described by authors |
| Year (costs) | Year of currency values presented (e.g. 2018 dollars) |
| Unit(s) of output | Choice of output measure (e.g. number of clients tested, number of facilities with testing available, etc.) |
| Sample size | Total number of, for example, facilities, individuals, tests |
| Timeframe for decision | Short-run (fixed inputs cannot be changed) and long-run (all inputs can be changed) (Kumaranayake, 2008) |
| Cost categories | Categorisation of costs as defined by author(s) |
| Economies/diseconomies of scale | How costs changed with scale of output and by how much. |
| Empirical results | Specific findings related to the costs of scaling-up (e.g. coefficients of scale) |
| Key drivers of costs identified | Key drivers of the costs of scaling-up (e.g. geography, population sub-group, type of providers, etc.) |
