# Supplementary material for: The costs of scaling up HIV and syphilis testing in low- and middle-income countries: a systematic review
Source: Health Policy Plan. 2021 Mar 9;36(6):939–54. doi: 10.1093/heapol/czab030 (PMC8227996; doi:10.1093/heapol/czab030)
Supplement: czab030_Supp [file czab030_supp.zip › Table 2.docx]

Table 2. List of appraisal checklist questions

| **Table 2.** Appraisal checklist | | |
| --- | --- | --- |
| **Standard of reporting costs** | | |
| **#1** | Was the research question(s) well defined? | (Yes/No/Partially addressed) |
| **#2** | Was the perspective of the cost estimation clearly stated? | (Yes/No/Partially addressed) |
| **#3** | Was the time horizon of sufficient length to capture the costs of the intervention at scale? | (Yes/No/Partially addressed) |
| **#4** | Did the study include relevant inputs in the cost estimation (i.e. consumables, human resources, equipment and infrastructure, and managerial practice) (Johns and Torres, 2005a)? | (Yes/No/Partially addressed) |
| **#5** | Were the methods for estimating the quantities of inputs clearly described? | (Yes/No/Partially addressed) |
| **#6** | Did the study clearly report the selection of data source(s) for the ‘units’ estimated in the cost per unit? | (Yes/No/Partially addressed) |
| **#7** | Was the sample size determined by the precision required for costing? If not, was the sample designed to be an accurate representation of the study population? | (Yes/No/Partially addressed) |
| **#8** | Did the study use relevant and appropriate discount, inflation, and currency conversion rates to enable cost adjustment over setting and time? | (Yes/No/Partially addressed) |
| **#9** | Did the study perform sensitivity analyses to characterise uncertainty associated with cost estimates? | (Yes/No/Partially addressed) |
| **#10** | Were cost estimates reported and communicated in a clear and transparent way? | (Yes/No/Partially addressed) |
| **Methodological quality of estimating costs at scale** | | |
| **#11** | Were average costs estimated for different levels of scale; and if so, did the study account for changes in input costs associated with increasing scale of the intervention? | (Yes/No/Partially addressed) |
| **#12** | Did the study quantify the relationship between average cost and scale? | (Yes/No/Partially addressed) |
| **#13** | Were fixed and variable costs analysed separately as scale increased? | (Yes/No/Partially addressed) |
| **#14** | Were factors other than scale, that could impact on cost, accounted for in the analysis? (e.g. scope, geography, target population, type of provider) | (Yes/No/Partially addressed) |
