# Supplementary material for: The costs of scaling up HIV and syphilis testing in low- and middle-income countries: a systematic review
Source: Health Policy Plan. 2021 Mar 9;36(6):939–54. doi: 10.1093/heapol/czab030 (PMC8227996; doi:10.1093/heapol/czab030)
Supplement: czab030_Supp [file czab030_supp.zip › Table 3.docx]

**Table 3.** Studies of the cost of scaling up HIV and syphilis testing

| **Table 3.** Studies of the cost of scaling up HIV and syphilis testing | | | | | | | | | |
| --- | --- | --- | --- | --- | --- | --- | --- | --- | --- |
| **Study** | **Intervention(s)** | **Country** | **Study population** | **Setting** | **Time horizon** | **Study design** | **Type of economic analysis (and ratio if applicable)** | **Data source (s)** | **Analytical approach to measure scale** |
| #1 (Shelley *et al.*, 2015) | Syphilis testing/ rapid syphilis test (RST)/point-of care (POC) | Zambia | Pregnant women  (age not specified) | ANC clinic | 5 months | Cross-sectional study | Cost analysis | Primary data collection | Empirical |
| #2 (Schackman *et al.*, 2007) | Syphilis testing, comparing:  #1 Syndromic surveillance/not POC  #2 Rapid plasma regain (RPR) for syphilis/not POC  #3 Rapid syphilis testing/POC | Haiti | Pregnant women  (age not specified) | ANC clinic | Not specified | Modelling | Cost-effectiveness analysis | Published literature/data | Modelling |
| #3 (Bautista-Arredondo *et al.*, 2018) | HIV voluntary and provider-initiated counselling and testing/HIV rapid test/not POC | Nigeria | General population  (age not specified) | Health facility | 6 months | Cross-sectional study | Cost analysis | Primary data collection | Econometric |
| #4 (Dandona, S. P. Kumar, *et al.*, 2008) | HIV voluntary counselling and testing/HIV rapid test/not POC | India | General population  (age not specified) | Health facility | 1 year | Cross-sectional study | Cost analysis | Primary data collection | Empirical |
| #5 (Galárraga *et al.*, 2017) | HIV testing and counselling/HIV rapid test/not POC | Kenya | General population  (age not specified) | Health facility | 22 months | Cross-sectional study | Cost analysis | Primary data collection | Econometric |
| #6 (Hontelez *et al.*, 2013) | HIV testing/type of test not specified/not POC, comparing:  #1 ART at CD4 count ≤ 350 cells/µl  #2 Universal testing and treatment | South Africa | General population  (aged 15 – 65 years old) | Not specified | Lifetime | Modelling | Cost-effectiveness analysis | Published literature/data | Modelling |
| #7 I(Ishikawa *et al.*, 2016) | HIV testing/HIV rapid test/not POC, comparing:  #1 the current coverage  #2 A focused approach  #3 A universal approach | Namibia, Kenya, Haiti, and Vietnam | Pregnant women  (aged 15 – 49 years old) | ANC clinic | 20 years | Modelling | Cost-effectiveness analysis | Published literature/data | Modelling |
| #8 (McConnel *et al.*, 2005) | HIV rapid voluntary counselling and testing/HIV rapid test/POC | South Africa | General population  (age not specified) | VCT clinic | 1 year | Cross-sectional study | Cost analysis | Primary data collection | Empirical |
| #9 (Ahaibwe and Kasirye, 2013) | HIV testing/HIV rapid test/not POC, comparing:  #1 The current coverage  #2 100% coverage of adult population | Uganda | General population  (adult 15 – 49 years old) | Stand alone, integrated w/ health facility, non- health facility and mobile VCT services | Lifetime | Modelling | Cost-effectiveness analysis | Published literature/data | Modelling |
| #10 (Alsallaq *et al.*, 2017) | HIV testing/HIV rapid test/not POC, comparing:  #1 HIV strategies focusing on youth (15 – 24 years old)  #2 HIV strategies focusing on adults (15+ year-old) | Kenya | Youth population  (aged 15 -24 years old) | Health facility | 20 years | Modelling | Cost-effectiveness analysis | Published literature/data | Modelling |
| #11 (Cambiano *et al.*, 2015) | HIV testing/HIV rapid test/not POC, comparing:  #1 Provider-delivered HIV testing and counselling  #2 HIV self-testing | Zimbabwe | General population  (aged 15 – 65 years old) | Not specified | 20 years | Modelling | Cost-effectiveness analysis | Published literature/data | Modelling |
| #12 (Cherutich *et al.*, 2018) | Assisted-partner service HIV testing (aPS)/rapid HIV test/not POC, comparing:  #1 Current coverage at 5%  #2 Scale-up to reach coverage of 50% | Kenya | General population  (age not specified) | Health facility and community-based health services | 5 years | Modelling | Cost-effectiveness analysis and budget impact analysis | Published literature/data | Modelling |
| #13 (Mwenge *et al.*, 2017) | HIV voluntary and provider-initiated counselling and testing/ rapid HIV test/not POC | Malawi, Zambia, and Zimbabwe | General population  (aged 15 – 49 years old) | Health facility | 1 year | Cross-sectional study | Cost analysis | Primary data collection | Econometric |
| #14 (Stuart *et al.*, 2018) | HIV testing/type of test not specified/not POC | South Africa | General population  (age not specified) | Not specified | 30 years | Modelling | Cost analysis | Published literature/data | Modelling |
| #15 (Tromp *et al.*, 2013) | HIV voluntary counselling and testing /HIV rapid test/not POC, comparing:  #1 current practice  #2 scaling-up to reach coverage 80% | Indonesia | Key populations (FSW, IDUs, higher-risk MSM, transgender, prisoner, clients of FSWs and partner IDUs) (age not specified) | Community-based VCT clinic | 20 years | Modelling | Cost-effectiveness analysis | Published literature/data | Modelling |
| #16 (Zhuang *et al.*, 2018) | HIV testing and treatment/type of test not specified/not POC, comparing:  #1 Current strategy  #2 Reached 90-90-90 target by 2020  # Reached 90-90-90 target by 2025 | China | MSM  (age not specified) | Health facility | 20 years | Modelling | Cost-effectiveness analysis | Published literature/data | Modelling |
| #17 (Dandona, S. G. P. Kumar, *et al.*, 2008) | HIV counselling and testing/HIV rapid test/not POC | India | Pregnant women  (age not specified) | Hospital and community health centre | 1 year | Cross-sectional study | Cost analysis | Primary data collection | Econometric |
| #18 (Dandona *et al.*, 2005) | HIV counselling and testing/HIV rapid test/not POC | India | General population  (age not specified) | VCT clinics | 1 year | Cross-sectional study | Cost analysis | Primary data collection | Econometric |
| #19 (Forsythe, 2002) | HIV voluntary counselling and testing/serial of HIV rapid testing/POC | Kenya | General population  (age not specified) | Health facility | A year | Cross-sectional study | Cost analysis | Primary data collection | Empirical |
| #20 (Granich *et al.*, 2009) | HIV testing/type of test not specified/not POC, comparing:  #1 Reference scenario  #2 Universal voluntary HIV testing and immediate ART | South Africa | General population  (age not specified) | Not specified | 42 years | Modelling | Cost-effectiveness analysis | Published literature/data | Modelling |
| #21 (Kasymova, Johns and Sharipova, 2009) | HIV testing and counselling/serial of HIV rapid test/not POC | Tajikistan | Youth population  (aged 15 – 25 years) | Youth friendly health services | 2 years | Cross-sectional study | Cost analysis | Primary data collection | Modelling |
| #22 (Kato *et al.*, 2013) | HIV testing type of test not specified/not POC, comparing:  #1 Reference scenario  #2 Targeted PTIT scenario  #3 Universal PTIT scenario | Vietnam | Key population: PWID, MSM, FSWs, MCF, IDU and low-risk women  (age not specified) | Health facility | 50 years | Modelling | Cost effectiveness analysis | Published literature/data | Modelling |
| #23 (Kumar *et al.*, 2006) | HIV testing and counselling/rapid HIV test/not POC, comparing:  #1 A universal testing  #2 A targeted testing | India | Pregnant women  (age not specified) | ANC clinic | Lifetime | Modelling | Cost-benefit analysis | Published literature/data | Modelling |
| #24 (Dandona *et al.*, 2009) | HIV voluntary counselling and testing/HIV rapid test/not POC | India | General population  (age not specified) | VCT clinic | 1 year | Cross-sectional study | Cost analysis | Primary data collection | Modelling |
| #25 (Mangenah *et al.*, 2019) | HIV self-testing (HIVST)/HIV rapid testing/not POC | Malawi, Zambia, and Zimbabwe | General population  (aged 15 – 59 years) | Community based distributing agent | 1 year | Cross-sectional study | Cost analysis | Primary data collection | Econometric |
| #26 (McCreesh *et al.*, 2017) | HIV self-testing/HIV rapid test/not POC, comparing  #1 current coverage  #2 Increased HIV testing (doubled) | Uganda | General population  (aged under 51 years) | Door-to-door community-based | 15-years | Modelling | Cost-effectiveness analysis | Published literature/data | Modelling |
| #27 (Minh *et al.*, 2012) | HIV voluntary counselling and testing/type of test not specified/not POC | Vietnam | General population  (age not specified) | Facility-based and freestanding VCT clinic | 4 months | Costing study | Cost analysis | Primary data collection | Modelling |
| #28 (Sharma *et al.*, 2016) | HIV home-based partner education and testing (HOPE)/HIV rapid test/not POC, comparing:  #1 Standard care (facility-based HIV testing)  #2 Adding HOPE to standard care | Kenya | Partner of pregnant women  (aged 0 – 59 years) | Home-based | 10-years | Modelling alongside randomised controlled trial | Cost-effectiveness analysis | Primary data collection | Modelling |
| #29 (Nelwan *et al.*, 2016) | HIV testing and counselling/HIV rapid test/not POC | Indonesia | Prisoner  (age not specified) | Outpatient clinic in prison | 3 years | Case-control study | Cost analysis | Primary data collection | Modelling |
| #30 (Luong Nguyen *et al.*, 2018) | HIV voluntary counselling and testing/HIV rapid test/not POC, comparing:  #1 Current testing coverage of 62%  #2 Scale up testing coverage to 90% | Kenya | General population  (age not specified) | Health facility | 20 years | Modelling | Cost-effectiveness analysis and budget impact analysis | Published literature/data | Modelling |
| #31 (Rely *et al.*, 2003) | HIV counselling and testing/HIV rapid test/not POC, comparing:  #1 Status quo (with 4% testing coverage)  #2 Increase in coverage to 85% | Mexico | Pregnant women  (age not specified) | Health facility | Lifetime | Modelling | Cost-effectiveness analysis | Published literature/data | Modelling |
| #32 (Tchuenche *et al.*, 2018) | Very early infant diagnosis (VEID)/DNA-PCR/not POC | Lesotho | HIV-exposed infants  (0 – 2 weeks) | Health facility | 1 year | Retrospective observational study | Cost analysis | Primary data collection | Modelling |
| #33 (Verstraaten *et al.*, 2017) | HIV voluntary counselling and testing/HIV rapid test/not POC | Indonesia | FSWs  (age not specified) | Mobile VCT services | 1 year |  | Cost analysis | Primary data collection | Modelling |
| #34 (Zang *et al.*, 2016) | HIV testing, comparing:  #1 One4All (include testing, counselling, CD4 results, and viral load)/HIV rapid test/POC  #2 Standard care/HIV rapid test+ western blot confirmatory test/POC | China | General population  (aged 15 – 64 years old) | Hospital | 1-,5-and 25-years | Modelling | Cost-effectiveness analysis alongside clustered randomized trial | Published literature/data | Modelling |
| #35 (Zhang *et al.*, 2015) | HIV testing/type of test not specified/not POC, comparing  #1 Status quo  #2 Reach universal coverage by 2015  #3 Reach universal coverage by 2017  #4 Reach universal coverage by 2022 | Thailand | MSM  (age not specified) | Health facility | 3-,5-and 10-years | Modelling | Cost-effectiveness analysis | Primary data collection | Modelling |
| Definitions: 90-90-90 target: 90% of all people living with HIV will know their HIV status, 90% of all people with diagnosed HIV infection will receive sustained antiretroviral therapy and 90% of all people receiving antiretroviral therapy will have viral suppression. ANC: antenatal care; aPS: assisted partner service; ART: antiretroviral treatment; CD4: cluster of differentiation 4; DNA-PCR: Deoxyribonucleic acid-polymerase chain reaction, a molecular diagnostic testing using DNA sequencing; FSWs: female sex workers; HIV: human immunodeficiency virus; HIVST: HIV self-testing; IDU: injected drug user; MSM: men who have sex with men; MCF: male clients of female sex workers; PMTCT: prevention mother-to-child transmission; POC: point-of-care; PTIT: HIV periodic testing and immediate treatment; PWID: people with injected drug; RDT: rapid diagnostic test for HIV; RNA: ribonucleic acid; RPR: rapid plasma regain; RST: rapid syphilis testing; VCT: HIV voluntary counselling and testing; | | | | | | | | | |
