# Supplementary material for: The costs of scaling up HIV and syphilis testing in low- and middle-income countries: a systematic review
Source: Health Policy Plan. 2021 Mar 9;36(6):939–54. doi: 10.1093/heapol/czab030 (PMC8227996; doi:10.1093/heapol/czab030)
Supplement: czab030_Supp [file czab030_supp.zip › Table 4.docx]

**Tables 4.** Studies that report on how costs of testing change with scale

| **Table 4.** Studies that report on how costs of testing change with scale | | | | | | | | | |
| --- | --- | --- | --- | --- | --- | --- | --- | --- | --- |
| **Ref** | **Definition of scaling-up** | **Year (costs)** | **Units of output** | **Sample size** | **Timeframe for decision** | **Authors’ categorisation of costs** | **Findings related to scale and cost** | **Results** | **Key drivers of costs identified*** |
| #1 Shelley et al, 2015 | Increase in geographic coverage | 2012 | Number of facilities | Pilot: 5 facilities in two districts  Scale-up: 4 facilities in two districts | Short run | **Incremental costs**  Start-up costs: personnel, per diems, conference hire, training equipment and supplies, and vehicle transport  Capital costs: vehicles and computers  Recurrent costs: personnel, supplies (syphilis testing, shared supplies, treatment), vehicle fuel and maintenance, quality assurance/control, and supervision | Diseconomies of scale | Average unit cost per woman tested USD 3.19 (pilot) and USD 11.16 (scale-up) | **Geography and infrastructure:** central level supervision and transport costs  **Managing the process of scaling-up:** quality assurance/control  **Other:** higher RST kit cost and lower RST uptake |
| #3 Bautista-Arredondo et al, 2018 | Increase in number of clients receiving a test | 2013 | Number of clients | 414 540 clients in 141 HIV testing and counselling facility | Short run | **Economic cost**  Personnel: staff salaries  Recurrent inputs and services: HIV testing kit and treatment  Capital: equipment (i.e. PCR machine, CD4 testing machine, refrigerator) and vehicles  Training: incl. opportunity costs of staff involved | Economies of scale | Average unit cost per client tested USD 30  Coefficient of scale** = - 0.44 | **Managing the process of scaling-up:** central-level financial decision, and task-shifting (i.e. reorganising human resource to delegate some tasks to less specialised health workers)  **Fixed costs:** the distribution of fixed cost to a larger number of people  **Other**: services integration, external supervision, and different level of service delivery |
| #4 Dandona et al, 2008 | Increase in number of clients receiving a test | 2006 | Number of clients | Pilot: 32 413 clients  Scale-up: 66 445 clients  17 hospital based VCT clinic | Short run | **Economic cost**  Personnel: staff payroll  Recurrent goods: HIV test kits, condoms, IEC (information, education, and communication) materials, medical supplies, and stationery  Recurrent services: staff training, local travel, building maintenance and utilities  Capital goods: furniture, medical and administrative equipment  Building: based on area-specific rentals | Economies of scale | Average unit cost per client tested USD 5.46 (pilot) and USD 3.3 (scale-up) | **Fixed costs:** the distribution of fixed cost to a larger number of people |
| #5 Galarraga et al, 2017 | Increase in number of clients receiving a test | 2011 | Number of clients per year | 237 160 clients in 56 sites HTC clinic | Short run | **Economic cost**  Personnel: staff and volunteer time  Recurrent supplies: HIV test kits, condoms  Recurrent operating costs: utilities and maintenance  Capital goods: equipment – purchase, maintenance, and replacement  Other inputs: administration, supervision, training | Economies of scale | Average cost per client tested is USD 7  Coefficient of scale **= - 0.18 | **Managing the process of scaling-up:** task shifting (i.e. using qualified lower level staff instead of physicians)  **Fixed costs:** the distribution of fixed cost to a larger number of people |
| #8 McConnel et al, 2005 | Increase in number of clients receiving a test | 2003 | Number of clients | 693 clients | Short run | **Economic and financial costs**  Personnel: Staff and volunteer time  Other recurrent goods and services: Staff and community training, campaign and publicity materials, utilities, stationary and donated HIV test kits, and condoms  Capital: Office equipment and building mortgage | Economies of scale | Average cost per VCT client tested is USD 161.03 (pilot) and USD 53.02 (scale-up) | **Fixed costs:** the distribution of fixed cost to a larger number of people |
| #13 Mwenge et al, 2017 | Increase in the total number of test kits distributed | 2016 | Number of test kits | A total of 7 735 test kits distributed in 54 HIV testing services units | Short run | **Economic and financial costs**  Capital costs: Buildings and storage, equipment, and vehicles  Recurrent costs: Personnel, training, HIV testing commodities, general supplies, facility-level operation, and waste management  Overhead costs: facility-level and HIV testing services (HTS) centre-level | Economies of scale | Average cost per client tested is USD 4.92 (Malawi), USD 4.24 (Zambia), and USD 8.79 (Zimbabwe)  Coefficient of scale**= not provided | **Fixed costs:** the distribution of fixed cost to a larger number of people  **Personnel:** staff salaries and training  **Other**: service integration and lack of demand |
| #17 Dandona et al, 2008 | Increase in number of clients receiving a test | 2006 | Number of clients | 125 073 clients in 16 PMTCT centres | Short run | **Economic and financial costs**  Rental: building and land  Personnel: Staff time  Capital goods: Furniture, medical and administrative equipment  Recurrent goods: HIV test kits, Nevirapine, disposable supplies, stationary and miscellaneous item  Recurrent services: Staff training, building maintenance and utilities, and waste disposal | Economies of scale | Average cost per client tested is USD 4.29 (pilot) and USD 1.61 (scale-up) | **Fixed costs:** the distribution of fixed cost to a larger number of people |
| #18 Dandona et al, 2005 | Increase in number of clients receiving a test | 2003 | Number of clients | 32 413 clients in 17 VCT clinics | Short run | **Economic cost**  Salaries: staff and volunteer time  Rentals: building and land  Capital goods: Furniture and medical equipment  Recurrent goods: HIV test kits, male condoms, IEC material, recurrent medical supplies, and stationery  Recurrent services: Staff training, building maintenance and utilities, and waste disposal | Economies of scale | Average cost per client tested is USD 5.46  Coefficient of scale** = - 0.83 | **Fixed costs:** the distribution of fixed cost to a larger number of people  **Other:** lack of demand |
| #19 Forsythe et al, 2002 | Increase in number of clients receiving a test | 1999 | Number of clients | 519 clients in three health centres | Short run | **Economic costs**  Labour costs: staff salaries  Materials and medication: HIV test kits, needles, syringes, and gloves  Equipment and furniture: no detail provided  Property and utilities: building rental value | Economies of scale | Average cost per client tested is USD 16  Coefficient of scale**= not provided | **Fixed costs:** the distribution of fixed cost to a larger number of people |
| #25 Mangenah et al, 2019 | Increase in the total number of test kits distributed | 2019 | Number of test kits | A total of 349 719 test kits distributed in 71 sites | Short run | **Economic and financial costs**  Start-up costs: training and community sensitization activities  Capital costs: building and storage, equipment, and vehicle  Recurrent costs: personnel, supplies, HIV self-test kits vehicle and building operation/maintenance, recurrent training, and waste management | Economies of scale | Average cost per kit distributed is USD 8.15 (Malawi), USD 16.42 (Zambia), and USD 13.84 (Zimbabwe)  Coefficient of scale**= not provided | **Fixed costs:** the distribution of fixed cost to a larger number of people |
| *****Categories for key drivers are summarised as geography and infrastructure, fixed costs, personnel, managing the process of scaling-up and other, as discussed by Benjamin Johns et al (Johns and Torres, 2005b).  **Coefficient of scale is a measure of association between average cost and level of scale. | | | | | | | | | |
