# Supplementary material for: The costs of scaling up HIV and syphilis testing in low- and middle-income countries: a systematic review
Source: Health Policy Plan. 2021 Mar 9;36(6):939–54. doi: 10.1093/heapol/czab030 (PMC8227996; doi:10.1093/heapol/czab030)
Supplement: czab030_Supp [file czab030_supp.zip › Table 5.docx]

Table 5. Results of the appraisal

| **Table 5.** Results of the appraisal | | | | | | | | | | | | | | |
| --- | --- | --- | --- | --- | --- | --- | --- | --- | --- | --- | --- | --- | --- | --- |
| **References** | **Costs** | | | | | | | | | | **Costs at scale** | | | |
|  | #1 Question(s) | #2 Perspective | #3 Time horizon | #4 Relevant inputs | #5 Methods for quantities | #6 Data source(s) | #7 Sample size | #8 Discount rate | #9 Sensitivity analysis | #10 costs reporting | #11 costs and scale | #12 quantification | #13 fixed & variable costs | #14 Factors other than scale |
| Shelley et al, 2015 | ✓ | ✓ | (✓) | ✓ | ✓ | X | (✓) | ✓ | ✓ | ✓ | (✓) | ✓ | ✓ | ✓ |
| Schackman et al, 2007 | ✓ | ✓ | X | (✓) | ✓ | ✓ | X | (✓) | ✓ | ✓ | (✓) | X | X | ✓ |
| Bautista-Arredondo et al, 2018 | ✓ | ✓ | ✓ | ✓ | ✓ | ✓ | (✓) | (✓) | X | ✓ | ✓ | ✓ | ✓ | ✓ |
| Dandona et al, 2008 | ✓ | (✓) | ✓ | ✓ | ✓ | ✓ | (✓) | ✓ | X | ✓ | (✓) | X | ✓ | X |
| Galarraga et al, 2017 | ✓ | X | ✓ | ✓ | ✓ | ✓ | (✓) | ✓ | X | ✓ | ✓ | ✓ | ✓ | ✓ |
| Hontelez et al, 2013 | ✓ | ✓ | ✓ | (✓) | (✓) | (✓) | (✓) | ✓ | ✓ | (✓) | (✓) | X | X | X |
| Ishikawa et al, 2016 | ✓ | ✓ | (✓) | (✓) | X | ✓ | X | (✓) | ✓ | (✓) | X | X | X | (✓) |
| McConnel et al, 2005 | ✓ | X | ✓ | ✓ | ✓ | ✓ | X | (✓) | X | ✓ | ✓ | X | ✓ | X |
| Ahaibwe et al, 2013 | ✓ | X | ✓ | X | X | X | (✓) | (✓) | ✓ | (✓) | X | X | X | X |
| Alsallaq et al, 2017 | ✓ | ✓ | (✓) | (✓) | (✓) | ✓ | (✓) | (✓) | ✓ | ✓ | X | X | X | X |
| Cambiano et al, 2014 | ✓ | ✓ | (✓) | (✓) | (✓) | ✓ | X | (✓) | ✓ | (✓) | X | X | X | (✓) |
| Cherutich et al, 2018 | ✓ | ✓ | ✓ | ✓ | ✓ | ✓ | (✓) | (✓) | ✓ | ✓ | (✓) | X | (✓) | ✓ |
| Mwenge et al, 2017 | ✓ | ✓ | ✓ | ✓ | ✓ | ✓ | (✓) | √ | ✓ | ✓ | ✓ | ✓ | ✓ | ✓ |
| Stuart et al, 2018 | ✓ | X | (✓) | (✓) | (✓) | (✓) | X | X | X | (✓) | (✓) | X | X | (✓) |
| Tromp et al, 2013 | ✓ | ✓ | ✓ | (✓) | ✓ | (✓) | (✓) | (✓) | √ | (✓) | X | X | (✓) | ✓ |
| Zhuang et al, 2018 | ✓ | ✓ | ✓ | (✓) | (✓) | X | X | ✓ | X | (✓) | (✓) | X | X | X |
| Dandona et al, 2008 | ✓ | (✓) | ✓ | ✓ | ✓ | ✓ | (✓) | ✓ | X | ✓ | ✓ | ✓ | ✓ | ✓ |
| Dandona et al, 2005 | ✓ | X | ✓ | ✓ | ✓ | ✓ | (✓) | ✓ | X | (✓) | (✓) | ✓ | ✓ | ✓ |
| Forsythe et al, 2002 | ✓ | ✓ | ✓ | (✓) | ✓ | ✓ | X | (✓) | X | ✓ | X | ✓ | ✓ | ✓ |
| Granich et al, 2009 | ✓ | X | (✓) | (✓) | (✓) | (✓) | X | X | X | (✓) | X | X | X | X |
| Kasymova et al, 2009 | ✓ | ✓ | ✓ | ✓ | ✓ | ✓ | (✓) | ✓ | X | ✓ | (✓) | X | (✓) | X |
| Kato et al, 2013 | ✓ | X | ✓ | (✓) | X | ✓ | (✓) | (✓) | X | ✓ | (✓) | X | X | ✓ |
| Kumar et al, 2006 | ✓ | √ | (✓) | (✓) | (✓) | (✓) | X | ✓ | ✓ | (✓) | (✓) | X | X | ✓ |
| Dandona et al, 2009 | ✓ | (✓) | ✓ | ✓ | ✓ | ✓ | (✓) | ✓ | ✓ | (✓) | (✓) | X | (✓) | ✓ |
| Mangenah et al, 2019 | ✓ | ✓ | ✓ | ✓ | ✓ | ✓ | (✓) | ✓ | ✓ | ✓ | ✓ | ✓ | ✓ | X |
| McCreesh et al, 2017 | ✓ | ✓ | ✓ | (✓) | X | ✓ | (✓) | (✓) | ✓ | (✓) | X | X | X | ✓ |
| Minh et al, 2012 | ✓ | ✓ | ✓ | (✓) | ✓ | ✓ | (✓) | ✓ | X | ✓ | X | X | (✓) | X |
| Monisha et al, 2016 | ✓ | ✓ | ✓ | ✓ | ✓ | (✓) | (✓) | ✓ | ✓ | ✓ | (✓) | X | (✓) | (✓) |
| Nelwan et al, 2016 | ✓ | ✓ | ✓ | (✓) | X | (✓) | X | X | X | (✓) | X | X | X | X |
| Nguyen et al, 2018 | ✓ | (✓) | ✓ | ✓ | (✓) | ✓ | (✓) | ✓ | ✓ | ✓ | X | X | (✓) | X |
| Rely et al, 2003 | ✓ | ✓ | (✓) | (✓) | X | (✓) | X | (✓) | ✓ | (✓) | X | X | X | (✓) |
| Tchuenche et al, 2018 | ✓ | (✓) | ✓ | ✓ | ✓ | ✓ | (✓) | ✓ | (✓) | ✓ | X | X | X | ✓ |
| Verstraaten et al, 2017 | ✓ | ✓ | ✓ | ✓ | ✓ | (✓) | (✓) | (✓) | ✓ | ✓ | X | X | (✓) | X |
| Zang et al, 2016 | ✓ | ✓ | ✓ | (✓) | (✓) | ✓ | (✓) | ✓ | ✓ | (✓) | (✓) | X | X | (✓) |
| Zhang et al, 2015 | ✓ | (✓) | ✓ | ✓ | ✓ | ✓ | (✓) | (✓) | X | ✓ | (✓) | ✓ | (✓) | ✓ |
|  | | | | | | | | | | | | | | |
|  | ✓ | Yes | | X | No | | (✓) | Partially addressed | | | | | | |
