# Supplementary figures and images for: The costs of scaling up HIV and syphilis testing in low- and middle-income countries: a systematic review
Source: Health Policy Plan. 2021 Mar 9;36(6):939–54. doi: 10.1093/heapol/czab030 (PMC8227996; doi:10.1093/heapol/czab030)

**Figure 1.** PRISMA flow diagram


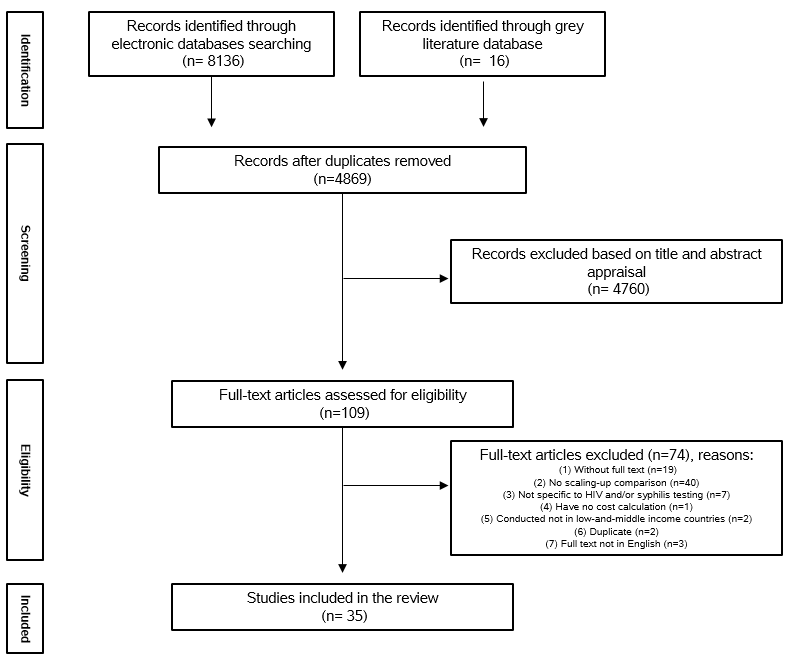

Supplement: czab030_Supp [file czab030_supp.zip › Figure black and white.docx]
